# Knockout of p75 neurotrophin receptor attenuates the hyperphosphorylation of Tau in pR5 mouse model

**DOI:** 10.18632/aging.102202

**Published:** 2019-09-03

**Authors:** Noralyn B. Mañucat-Tan, Lin-Lin Shen, Larisa Bobrovskaya, Mohammed Al-hawwas, Fiona H. Zhou, Yan-Jiang Wang, Xin-Fu Zhou

**Affiliations:** 1 School of Pharmacy and Medical Sciences, Sansom Institute for Health Research, University of South Australia, Adelaide 5000, Australia; 2 Department of Neurology and Center for Clinical Neuroscience, Daping Hospital, Third Military Medical University, Chongqing 400042, China

**Keywords:** Tau hyperphophorylation, pR5, p75 ^NTR^, tauopathy, neurotrophin

## Abstract

p75 neurotrophin receptor (p75^NTR^) has been implicated in Alzheimer’s disease (AD). However, whether p75^NTR^ is involved in Tau hyperphosphorylation, one of the pathologies observed in AD, remains unclear. In our previous study, the extracellular domain of p75^NTR^ blocked amyloid beta (Aβ) toxicity and attenuated Aβ-induced Tau hyperphosphorylation. Here we show that, in the absence of Aβ, p75^NTR^ regulates Tau phosphorylation in the transgenic mice with the P301L human Tau mutation (pR5). The knockout of p75^NTR^ in pR5 mice attenuated the phosphorylation of human Tau. In addition, the elevated activity of kinases responsible for Tau phosphorylation including glycogen synthase kinase 3 beta; cyclin-dependent-kinase 5; and Rho-associated protein kinase was also inhibited when p75^NTR^ is knocked out in pR5 mice at 9 months of age. The increased caspase-3 activity observed in pR5 mice was also abolished in the absence of p75^NTR^. Our study also showed that p75^NTR^ is required for Aβ- and pro-brain derived neurotrophin factor (proBDNF)-induced Tau phosphorylation, *in vitro*. Overall, our data indicate that p75^NTR^ is required for Tau phosphorylation, a key event in the formation of neurofibrillary tangles, another hallmark of AD. Thus, targeting p75^NTR^ could reduce or prevent the pathologic hyperphosphorylation of Tau.

## INTRODUCTION

Aggregated Tau helical filaments, referred to as neurofibrillary tangles (NFTs), contribute to the neurodegeneration in Alzheimer’s disease (AD) [1]. Tau pathology has also been implicated in other neurodegenerative diseases such as frontotemporal dementia (FTD) [2], progressive supranuclear palsy, Parkinson’s disease, Huntington’s disease, and Pick’s disease [3–6]. Tau is mainly found in the axons of neurons, and at low levels in glial cells, where it regulates the assembly of microtubules, the cytoskeleton reorganization and, the retrograde/anterograde transport of cargo through Tau’s interaction with dynein and kinesin [3, 7]. The phosphorylation and dephosphorylation process of Tau, and the amount of Tau phosphorylated at different sites can all contribute to the physiological and the pathological functions of Tau [3]. When Tau is hyperphosphorylated by various kinases, it misfolds and forms paired helical filaments, which eventually aggregate into NFTs. The level of NFTs in the brain of AD patients positively correlates with cognitive impairment while the presence of Tau mutations has been implicated in neuronal dysfunction [8]. Prominent kinases that are often implicated in other diseases have been reported to phosphorylate Tau and these include microtubule affinity-regulating kinases [9], glycogen synthase 3 beta (GSK3β), cyclic adenosine monophosphate (AMP)-dependent protein kinase A (PKA), cyclin-dependent protein kinase 5 (Cdk5) [3, 10–12], rho-kinase (ROCK) [13], and c-Jun N-terminal kinase (JNK) [14–16]. There is evidence showing Aβ mediates Tau phosphorylation induced by the interaction between PKA and JNK [17]. The inhibition of phosphatidylinositol kinase-3 (PI3K) also leads to the activation of GSK3β, inducing Tau hyperphosphorylation [18–21].

Several studies have shown that the accumulation of Aβ enhances Tau pathology while the excess of the latter shows no effects on Aβ toxicity, thus indicating that Aβ is upstream of Tau signaling [22–24]. However, the mechanism of how Aβ drives Tau pathology remains unclear. In addition, neurotrophins, proneurotrophins and their receptors can have an effect on Tau phosphorylation in different ways. In nerve growth factor (NGF)-deprived PC12 cells, Tau phosphorylation at Ser202 detected by monoclonal antibody AT8 is increased compared to non-deprived cells [25, 26] suggesting that a lack of trophic support may also lead to pathologic Tau phosphorylation. p75^NTR^ is a receptor belonging to a larger family of tumour necrosis factor receptors. Neurotrophins and proneurotrophins bind to p75^NTR^ and function in cell survival and apoptosis, respectively [27, 28]. p75^NTR^ is also reported to bind Aβ monomer [28]. The increased colocalization of p75^NTR^ with hyperphosphorylated Tau in the neurons found in AD brain further supports the role of neurotrophins in AD [29]. One of the proneurotrophins, that is known to induce neuronal apoptosis via p75^NTR^ is the pro-brain derived neurotrophic factor (proBDNF)_._ proBDNF in complex with another protein was also reported to co-localize with Tau in the axons and soma of neurons [30], thus proBDNF may also potentially regulate Tau phosphorylation via p75^NTR^. Inhibiting p75^NTR^ with LM11A-31 a small molecule p75^NTR^ ligand, has been found to reduce Aβ-induced Tau hyperphosphorylation and misfolding [31, 32]. Moreover, we have previously shown that the treatment of AD mice with the peptide containing the extracellular domain of p75^NTR^ fused to human Fc region (p75ECD-Fc) reduced Tau hyperphosphorylation and Aβ plaque formation and reversed cognitive impairments [33]. Although these findings suggest that p75^NTR^ has a role in AD pathology, it’s role in Tau hyperphosphorylation in AD needs further investigation.

In this study, we aimed to investigate the changes in Tau phosphorylation and the kinases involved after deletion of p75^NTR^ using a Tauopathy mouse model, pR5. Transgenic pR5 mice bear the human FTD Tau mutation P301L with Parkinsonism linked to chromosome 17, which results in Tau hyperphosphorylation and the formation of abnormal Tau filaments and in the absence of amyloid pathology [34, 35]. This makes pR5 mice an ideal model to elucidate the role of p75^NTR^ in Tauopathy and in Aβ-induced-Tau phosphorylation. By knocking out p75^NTR^ in pR5 mouse model we generated a new model, pR5^p75-/-^(pR75KO) for our study. We have found that the full-length p75^NTR^ is required for the hyperphosphorylation of Tau *in vivo* and *in vitro*. The deletion of p75^NTR^ also deactivated several kinases that mediate Tau phosphorylation such as GSK3β, Cdk5 and ROCK. We propose that p75^NTR^ is a potential regulator of Tauopathy and is required for Aβ-induced Tau hyperphosphorylation.

## RESULTS

### Knockout p75^NTR^ in pR5 mice reduced Tau staining in the brain

**Figure 1:**
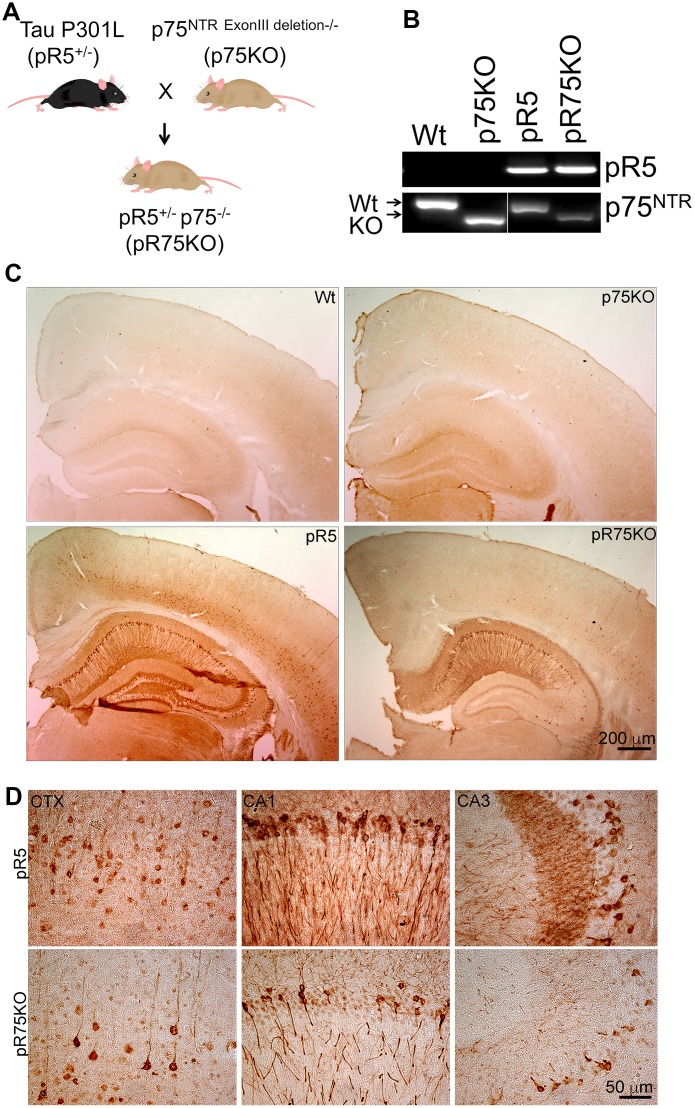
**Knock out of p75NTR in pR5 mice attenuated transgenic Tau protein staining in neurons.** (**A**) pR5 mice is cross bred with p75KO mice to generate pR75KO mice. (**B**) PCR confirmation of transgenic tau and p75^NTR^ knockout. (**C**) Transgenic Tau, detected using human-specific Tau and pTau antibody HT7, Scale bar = 200 μm. (**D**) Transgenic Tau expression in pR5 was much weaker in pR75KO mice in the cortex (CTX) and hippocampal regions CA1 and CA3 at higher magnification, Scale bar = 50 μm.

**Figure 2:**
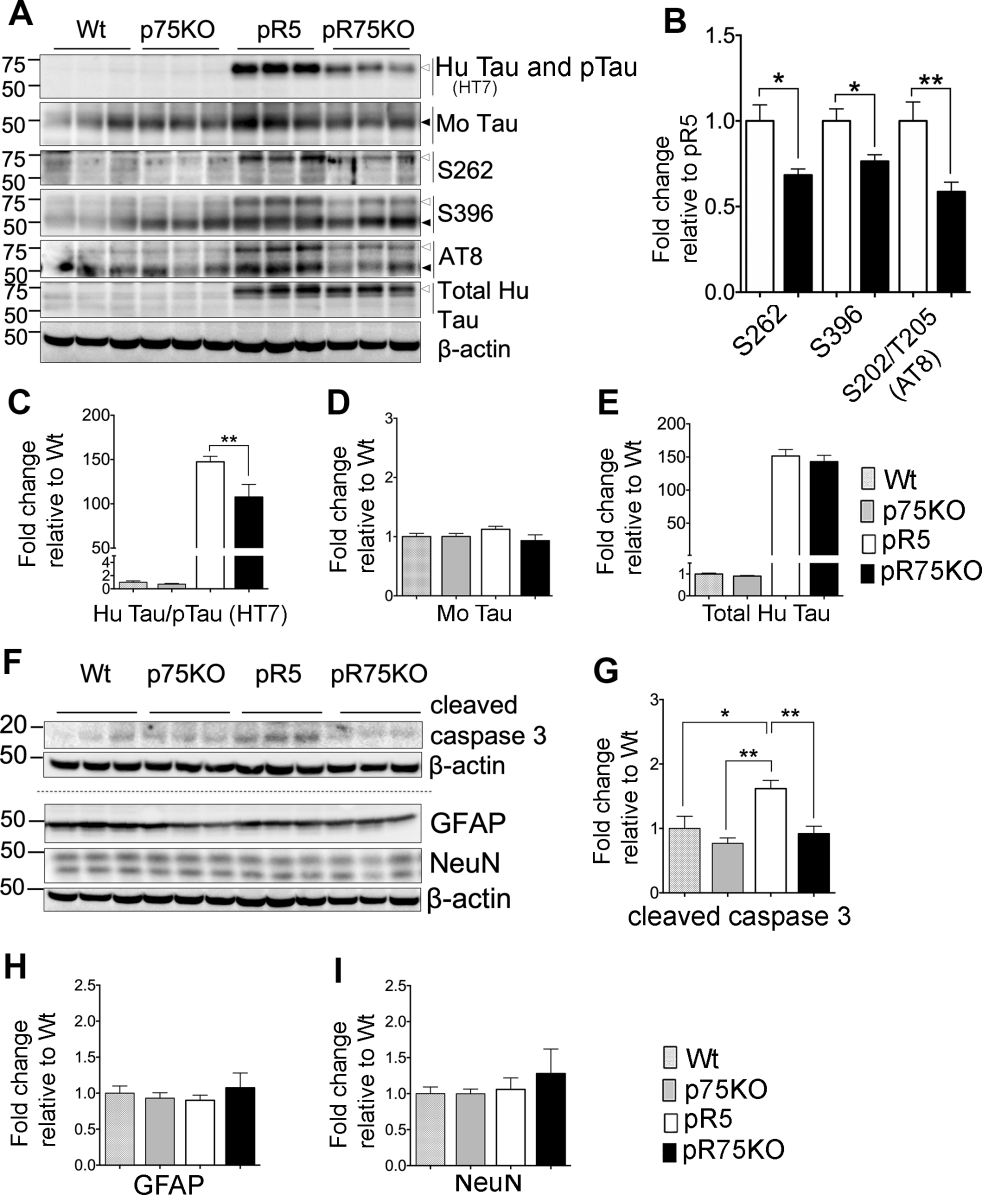
**Knock out of p75^NTR^ in pR5 mice attenuated human Tau phosphorylation at 6 months.** (**A**) Protein blots for total Tau, and phosphorylated Tau probed at 75 kDa for human and 50 kDa for mouse protein bands in the forebrains of Wt, p75KO, pR5 and pR75KO mice at 6 months. (**B**) Protein band intensity quantification of phosphorylated human Tau at S262, S396 and S202/T205 (AT8) in pR5 and pR75KO mice normalised with total human Tau and expressed as fold change relative to pR5. Protein band intensity quantification of total human Tau and pTau detected by HT7 (**C**), total mouse Tau detected by Tau5 (**D**), and total human Tau detected by sheep-anti human Tau (**E**) normalised to β-actin and expressed as fold change relative to Wt. (**F**) Protein blots of cleaved caspase-3, glial fibrillary acidic protein (GFAP) neuronal nuclei (NeuN). Protein band intensity quantification of cleaved caspase-3 (**G**), GFAP (**H**), and NeuN (**I**) normalised with their respective total β-actin and expressed as fold change relative to Wt. Data are represented as the mean ± SEM, n=6. Statistical comparisons were performed using one-way ANOVA and Tukey’s test. For human pTau, two-tailed unpaired t-test was used to compare pR5 and pR75KO mice. Statistical significance: *P<0.05, **P<0.01.

In order to examine the role of p75^NTR^ in Tau hyperphosphorylation, we crossed pR5 Tauopathy mice carrying the human Tau P301L mutation with p75KO mice which have a deletion of exon III of p75^NTR^ to obtain pR5^p75-/-^ (pR75KO) (Figure 1A). We selected pR75KO mice based on the genotyping results indicating the presence of the human Tau DNA band and p75^NTR^ DNA band while the wild type (Wt) p75 exon III DNA band was not detected (Figure 1B). In addition, using immunohistochemistry we confirmed the absence of p75^NTR^ protein expression in the substantia nigra of p75KO and pR75KO mice compared to Wt and pR5 mice which still expressed the p75^NTR^ exon III DNA and protein in the brain (Supplementary Figure 1). We also showed that human Tau is expressed in neurons of the cortex and hippocampal regions of the brain of only pR5 and the new transgenic pR75KO mice (Figure 1C, 1D). These results confirmed that we have successfully generated the pR5 Tauopathy model with p75^NTR^ deletion. We found that there were substantially fewer neurons which stained positive for human Tau using the anti-human Tau HT7 antibody in the brain of pR75KO mice (Figure 1D). The western blot with the same HT7 antibody which recognizes both human Tau and phosphorylated Tau further confirmed this observation (Figure 2C). To further examine whether the reduction in human Tau staining is a result of attenuated Tau phosphorylation or total Tau protein expression, we used different antibodies specific for Tau phosphorylated at various sites and an antibody against total human Tau.

### Attenuated phosphorylation of human Tau in pR75KO mice at 6 months of age

We compared the transgenic human Tau including phosphorylated Tau in the forebrains of 6 months old Wt, p75KO, pR5 and pR75KO mice using western blot analysis (Figure 2A). The levels of phosphorylation of Tau at different sites known to occur in FTDP-17 [36] such as Ser262 (S262), Ser396 (S396) and Ser202/Thr205 (AT8) (S202/T205 (AT8)) were detected in all mouse strains. We compared the change in phosphorylated human Tau (pTau) at 75 kDA in pR5 and pR75KO mice normalized against levels of total human Tau. We found that the levels of S262 (p=0.0102), S396 (p=0.0149) and S202/T205 (AT8) (p=0.0076) were significantly attenuated in pR75KO mice compared to pR5 mice (Figure 2B). Since total human Tau when normalized against β-actin had no change (Figure 2E; n=6, p=0.8070; one-way ANOVA, Tukey’s post-hoc test) in pR75KO mice when compared to pR5, these data suggest that p75^NTR^ mediates the phosphorylation of human Tau rather than the synthesis of human Tau protein. Furthermore, the hyperphosphorylation of human Tau in pR5 transgenic mice did not significantly affect the endogenous expression of total mouse Tau (Figure 2D) and mouse Tau phosphorylation (Supplementary Figure 2) at 6 and 9 months old animals. Deletion of p75^NTR^ in pR5 mice did not change total mouse Tau expression (Figure 2D) or the phosphorylation of mouse Tau at S396 and S202/T205 (AT8) (Supplementary Figure 2). This indicates Tau hyperphosphorylation is only occurring in the human protein as a result of the P301L Tau mutation.

### Reduction of caspase activity in pR75KO mice at 6 months of age

In the P301L mouse model, Tau mutation activates calpain, a protein reported to directly or indirectly activate caspase-3, thereby mediating the hyperphosphorylation of Tau [37]. We found that knocking out p75^NTR^ can reduce the protein level of active caspase-3, shown here as cleaved caspase-3 (Figure 2F). The level of cleaved-caspase-3 was elevated in pR5 mice compared to Wt (p=0.0196) and p75KO (p=0.0013) and reduced in pR75KO mice (p=0.0075) (Figure 2G). Thus, the knockout of p75^NTR^ is inhibiting the increase in caspase-3 activity caused by human Tau mutation in the pR75KO model. There was no evidence of gliosis, measured by GFAP, or neuronal loss, measured by NeuN, as a result of the Tau mutation and knockout of p75^NTR^ as shown by western blotting (Figure 2A, 2H, 2I) and IHC (Supplementary Figures 3–4). Therefore, it is hard to conclude based on the increased caspase-3 activity alone that hyperphosphorylation of Tau in pR5 mice resulted in apoptosis. These results are consistent with the previous findings showing that gliosis and significant neuronal loss in this P301L mouse model become evident only from 10 months of age [38, 39], thus in this transgenic mice at 6 or 9 months of age examined here, we did not observe such changes.

### Reduction of the activity of protein kinases in pR75KO mice involved in the phosphorylation of human Tau at 6 months of age

**Figure 3:**
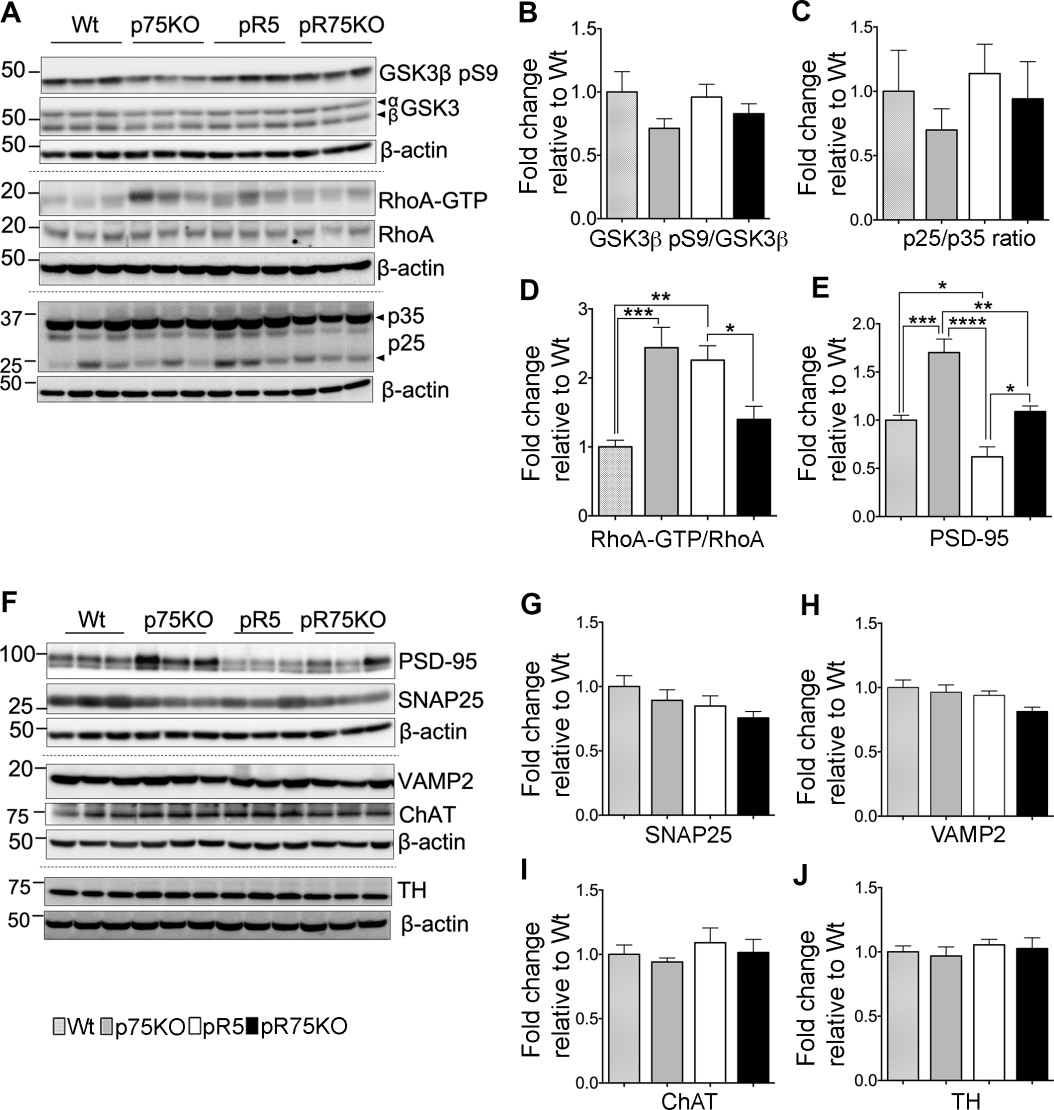
**Synaptic proteins, neuronal markers and Tau kinase activity in pR75KO at 6 months.** (**A**) Protein blots of kinases involved in Tau phosphorylation, GSK3β, RhoA and Cdk5 activators, p35 and p25 proteins in the forebrain of Wt, p75KO, pR5, and pR75KO mice. Protein band intensity quantification of inactive GSK3:GSK3β pS9 normalised with total GSK3β (**B**), Cdk5 activators, p25/p35 ratio (**C**), and active RhoA-GTP normalised with total RhoA (**D**). All band intensities showing B-D are expressed as fold change relative to Wt. F) Protein blots of post-synaptic protein, PSD-95 and pre-synaptic proteins, SNAP25 and VAMP2, tyrosine hydroxylase (TH) and choline acetyl transferase (ChAT). Protein band intensity quantification of PSD-95 (**E**), SNAP25 (**G**), VAMP2 (**H**), choline acetyl transferase (ChAT) (**I**), and tyrosine hydroxylase (TH) (**J**) normalised with total β-actin of respective blot and expressed as fold change relative to Wt. Data are represented as the mean ± SEM, n=6. Statistical comparisons were performed using one-way ANOVA and Tukey’s test. Statistical significance: *P<0.05, **P<0.01, ***P<0.001, ****P<0.0001.

Tau is phosphorylated by GSK3β, ROCK and Cdk5 [10, 12, 13, 40]. To determine whether p75^NTR^ is important for the phosphorylation of Tau mediated by these kinases, we probed the expression levels of GSK3β, RhoA and Cdk5 activators, p25 and p35 species, in the half-brain homogenates of 6 months old animals (Figure 3A). The protein levels of phosphorylated GSK3β at Ser9 (GSK3β pS9) in pR5 and pR75KO mice did not show clear changes at 6 months of age (Figure 3B). The enzymatic activity of Cdk5 is regulated by its activators, p35 and p39 [41]. Under neurotoxic conditions, p35 is cleaved by calpain to generate a 25 kDa fragment, referred to as p25 [41]. An increased ratio of p25 to p35 has been linked to neurodegeneration in AD and Tauopathy [41, 42]. Therefore, we measured the protein levels of p25 and p35 and found that the p25/p35 ratio was not significantly altered in pR5 mice and pR75KO mice (p>0.05) at this age (Figure 3C). We next detected the levels of total RhoA and its active form RhoA-GTP. The conversion of RhoA from inactive state (GDP-bound) to active state (GTP-bound) mediated by GTP binding also activates several downstream effectors including ROCK [43]. Activated ROCK could directly phosphorylate Tau [13, 44]. In this study, we found that the levels of RhoA-GTP were significantly higher in pR5 compared to Wt mice (p=0.0021) and attenuated in pR75KO mice (p=0.0411) compared to pR5 mice while p75KO had slightly higher level than pR5 on the blot but did not reach statistical significance (Figure 3D). These data suggest that ROCK is downstream of p75^NTR^ signaling [32, 45], and could potentially phosphorylate Tau through p75^NTR^.

### Synaptic proteins and neuronal markers are differentially expressed in mice strains at 6 months of age

The levels of presynaptic proteins, SNAP25 and VAMP2 were determined by western blot analysis in 6 months old mice. SNAP25 and VAMP2 protein expression were not altered by the transgenic strains compared to Wt mice (Figure 3F–3H). The post-synaptic protein PSD-95 was significantly elevated in p75KO compared to Wt (p=0.0002). However, PSD-95 protein expression in pR5 was suppressed compared to Wt (p=0.0484) and p75KO (p<0.0001). The deletion of p75^NTR^ in pR5 mice elevated PSD-95 protein expression in pR75KO mice (p=0.0018) to levels comparable to Wt mice. It is interesting to note that the significant difference in PDS-95 expression between p75KO and pR75KO (p=0.0011) is due to the human Tau P301L mutation. These results suggest that, p75^NTR^ may be a negative regulator of post-synaptic protein, PSD-95. A previous study showed that p75^NTR^ is highly expressed in protein fraction from mouse hippocampus that was also rich in PSD-95 [46]. We have yet to show how the role of p75^NTR^ influences PSD-95 transcription or post-translational modification in pR5 mice. Cholinergic degeneration is associated with cognitive decline in AD and FTD [47] but western blot analysis of cholinergic neuron marker, choline-acetyl transferase (ChAT), in pR5 and pR75KO mice, showed no changes (Figure 3I). pR5 mice did not show any reduction in ChAT levels similar to a previous finding [47]. We also checked the levels of tyrosine hydroxylase (TH), which is expressed in dopaminergic neurons in the substantia nigra and striatum [48]. Loss of TH-positive neurons is a characteristic of a severe form of FTD present in K396I Tau mutant mice [49]. We did not observe any difference in TH expression levels in all strains (Figure 3J), similar to another study that examined TH protein changes in wild type and p75KO mice [50]. At 6 months of age, knocking out of p75^NTR^ increased the levels of post-synaptic proteins, like PSD-95 but had no impact on presynaptic proteins or the population of cholinergic and dopaminergic neurons.

### Reduction of Tau phosphorylation, and kinase activities in pR75KO mice at 9 months of age

**Figure 4:**
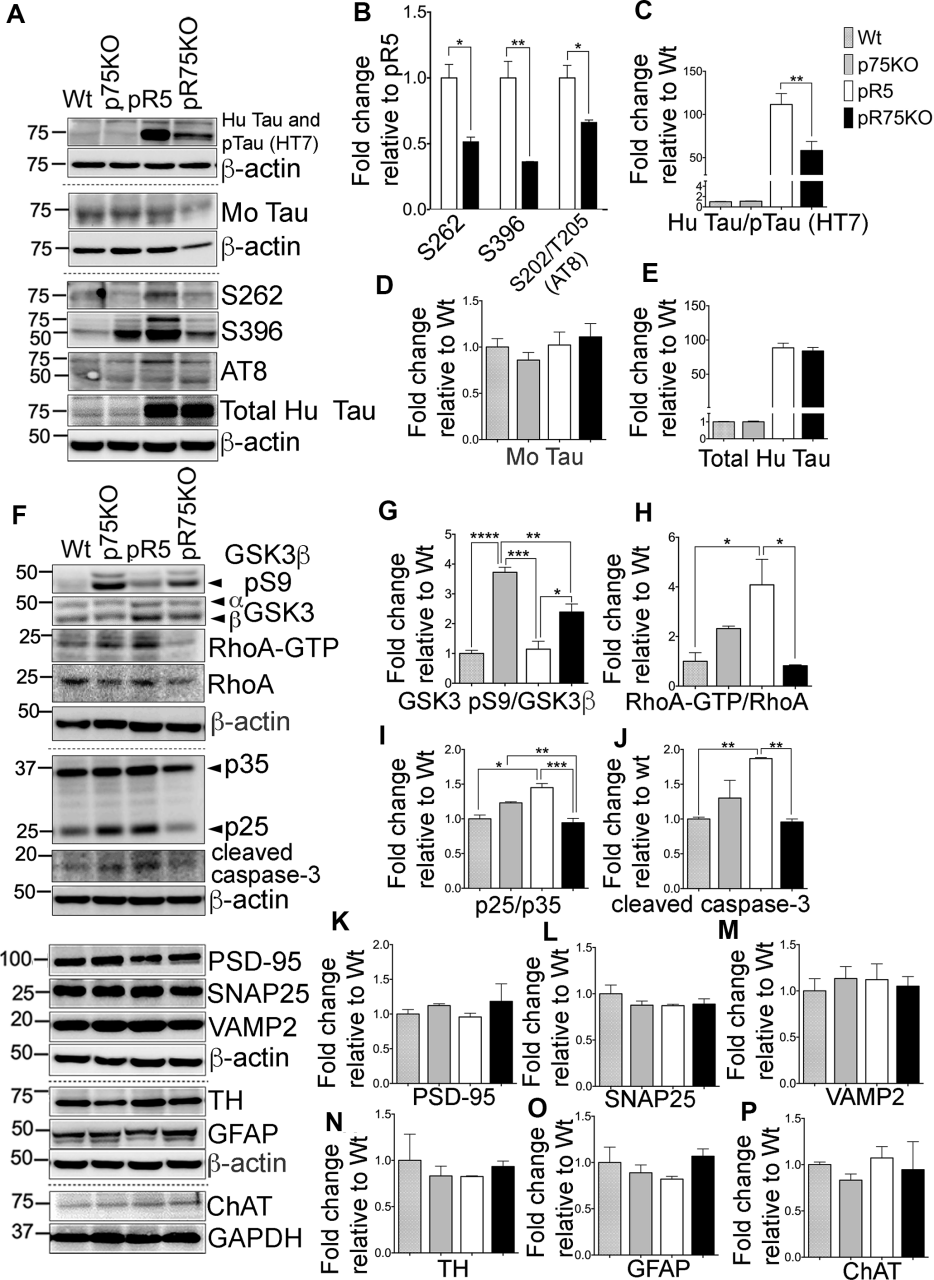
**Knock out of p75^NTR^ attenuated Tau hyperposphorylation and the elevated Tau kinases and caspase-3 activities observed in pR5 mice with P301L Tau at 9 months.** (**A**) Protein blots of phosphorylated and non-phosphorylated human Tau in in the forebrain of Wt, p75KO, pR5, and pR75KO mice. (**B**) Protein band intensity quantification of phosphorylated human Tau at sites S262, S396 and S202/T205 (AT8) normalised to the total human Tau and expressed as fold change relative to pR5. Protein band intensity quantification of total human Tau and pTau detected by HT7 (**C**), total mouse Tau detected by Tau5 (**D**), and total human Tau detected by sheep-anti human Tau (**E**) normalised to β-actin and expressed as fold change relative to Wt. (**F**) Protein blots of kinases involved in Tau phosphorylation, GSK3, RhoA and Cdk5-activators, p25 and p35 proteins in the forebrain of Wt, p75KO, pR5, and pR75KO mice; of cleaved caspase-3; and of post-synaptic protein, PSD-95 and pre-synaptic proteins, SNAP25 and VAMP2, GFAP, TH ChAT. Protein band intensity quantification of inactive GSK3: GSK3β pS9 normalised with total GSK3β (**G**), active RhoA-GTP normalised with total RhoA (**H**), and Cdk5 activators, p25/p35 ratio (**I**). All band intensities showing (**G**–**I**) are expressed as fold change relative to Wt. Protein band intensity quantification of cleaved caspase-3 levels (**J**), PSD-95 (**K**), SNAP25 (**L**), VAMP2 (**M**), TH (**N**), GFAP (**O**), ChAT (**P**) normalized with their respective β-actin and expressed as fold change relative to Wt. Data are represented as the mean ± SEM, n=3. Statistical comparisons were performed using one-way ANOVA and Tukey’s test. For human pTau, two-tailed unpaired t-test was used to compare pR5 and pR75KO mice Statistical significance: *P<0.05, **P<0.01, ***P<0.001, ****P<0.0001.

To determine if the reduction in phosphorylated Tau levels and kinase activities were reflected in older animals, we also investigated the protein levels of the kinases and synaptic markers in half-brain homogenates of 9 months old mice by western blot analysis (Figure 4A). Consistent with the results in 6 months old mice, Tau hyperphosphorylation at sites S262 (p=0.0111), S396 (p=0.0072) and S202/T205 (AT8) (p=0.0255) were all significantly reduced in pR75KO mice (Figure 4B) compared to pR5 mice. Again, using anti human Tau (HT7) which recognizes phosphorylated and non-phosphorylated Tau (Figure 4C) we found the band intensity was weaker in pR75KO mice compared to pR5 mice (p=0.0077). However, using specific antibodies against total mouse Tau (Figure 4D) and total human Tau (Figure 4E), we found the intensities were unaffected. Similar to 6 months old animals, phosphorylated mouse Tau at S396 and S202/T205 (AT8) were also unaffected in 9 months old animals (Supplementary Figure 2C–2D).

Inactive GSK3β (pS9) levels were significantly higher in p75KO (p=0.0001) and pR75KO mice (p=0.0142) (Figure 4F, 4G) compared to pR5 mice, suggesting that GSK3β is involved in Tau hyperphosphorylation in 9 months old pR5 mice. RhoA-GTP was significantly higher in pR5 mice compared to Wt (p=0.0171) and pR75KO (p=0.0126) (Figure 4F, 4H). The same reduction in p25/p35 ratio was observed in Wt (p=0.0181) and p75KO (p=0.0322) and pR75KO mice (p=0.0171) compared to pR5 mice (Figure 4F, 4I). These results confirmed that p75^NTR^ functions in the hyperphosphorylation of Tau during aging possibly through the activation of kinases such as GSK3β, ROCK and Cdk5. Similarly, the level of cleaved caspase-3 was also elevated in aged pR5 mice (p=0.0066) compared to Wt mice (Figure 4F, 4J) while it was significantly reduced in aged pR75KO mice compared to pR5 mice (p=0.0049). In the older animals tested, p75^NTR^ did not seem to influence presynaptic and post-synaptic proteins. SNAP25 and VAMP2 remained unchanged in all strains (Figure 4F, 4L, 4M). Although there is a subtle increase in PSD-95 in pR75KO mice, the increase remained insignificant (Figure 4F, 4K). TH, GFAP and ChAT remained unchanged in 9 months old animals similar to 6 months old animals (Figure 4F, 4N–4P). These results provide further evidence that the presence of p75^NTR^ could promote the pathways that lead to the phosphorylation of Tau including activation of kinases, such as GSK3β, RhoA and Cdk5, and caspase-3

### ProBDNF- and Aβ-induced Tau hyperphosphorylation requires p75^NTR^

**Figure 5:**
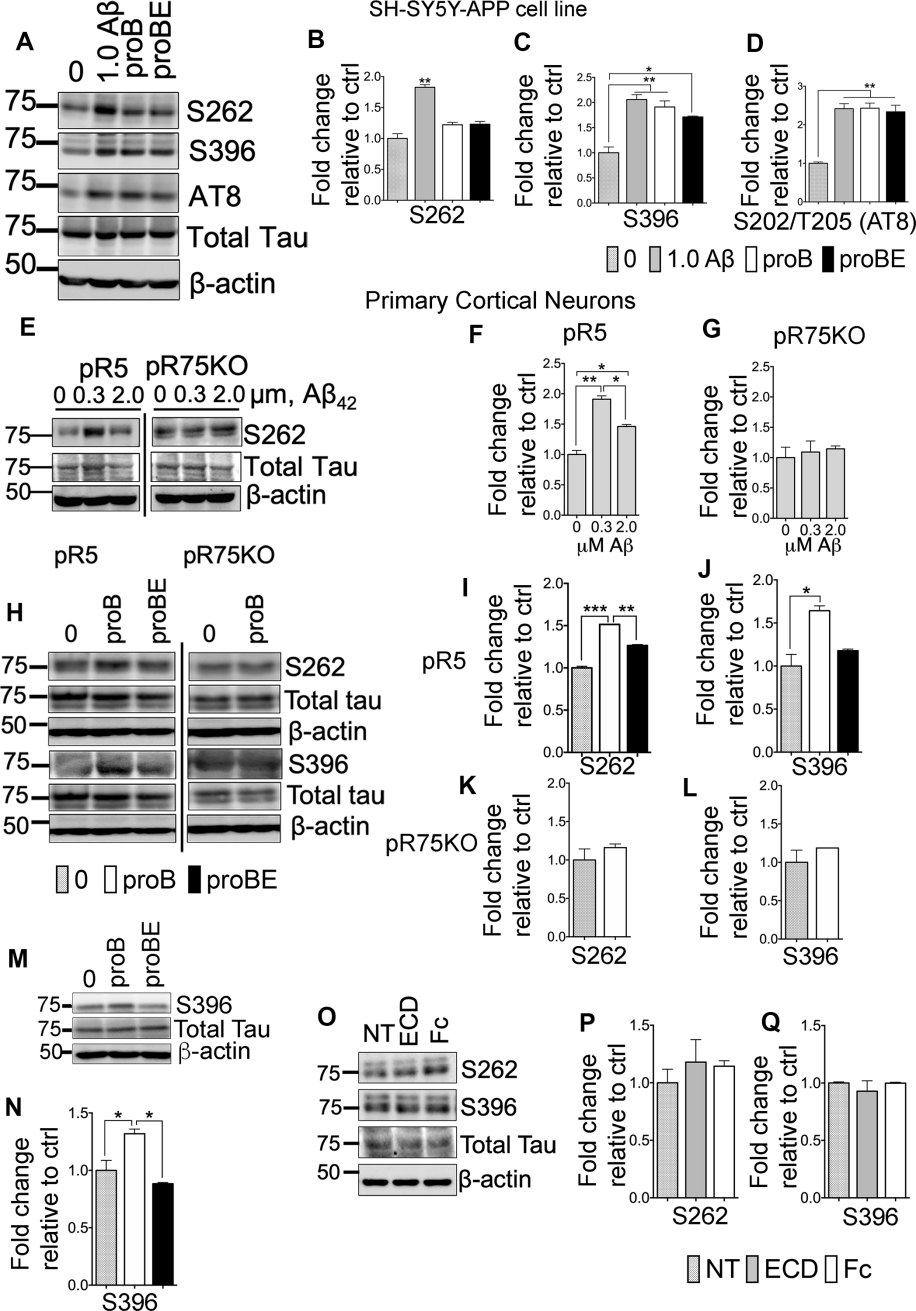
**p75^NTR^ ligands, Aβ and pro-BDNF, induced Tau hyperphosphorylation of neurons *in vitro*.** (**A**) Protein blots of phosphorylated human Tau at sites S262, S396 and S202/T205 (AT8) in SH-SY5Y-APP cell. Treatments were control (0), Aβ_42_ (1.0 μM)_,_ proBDNF (30 ng/mL, proB), and proBDNF (30 ng/mL,) with p75ECD-Fc (10 μg/mL) (proBE). Protein band intensity quantification of phosphorylated human Tau at S262 (**B**), S396 (**C**) and S202/T205 (AT8) (**D**) in SH-SY5Y-APP cell line normalised with total human Tau and expressed as fold change relative to non-treated control (0). Data are represented as the mean ± SEM, n=3. (**E**) Protein blots of phosphorylated human Tau at site S262 in primary cortical neurons from pR5 and pR75KO mice treated with different concentrations of Aβ_42_ (0, 0.3, 2.0 μM). Protein band intensity quantification of phosphorylated human Tau at S262 in neurons from pR5 (**F**) and pR75KO (**G**) mice normalized with total human Tau and expressed as fold change relative to non-treated control (0). Data are represented as the mean ± SEM. Experiment was done in 3 replicates, each replicate has n=12 animals. (**H**) Protein blots of phosphorylated human Tau at sites S262 and S396 in primary cortical neurons frompR5 and pR75KO mice treated with proB and proBE. Protein band intensity quantification of phosphorylated human Tau at S262 and S396 in neurons from pR5 (**I**, **J**) and pR75KO (**K**, **L**) mice normalized with total human Tau and expressed as fold change relative to non-treated control (0) Data are represented as the mean ± SEM. Experiment was done in 3 replicates, each replicate has n=12 animals. (**M**) Protein blots of phosphorylated human Tau at site S396 in primary cortical neurons from Wt mice treated with proB and proBE. (**N**) Protein band intensity quantification of phosphorylated human Tau S396 in Wt mice normalized with total human Tau and expressed as fold change relative to non-treated control (0) Data are represented as the mean ± SEM. Experiment was done in 3 replicates, each replicate has n=12 animals. (**O**) Protein blots of phosphorylated human Tau at sites S262 and S396 in primary cortical neurons of Wt mice treated with p75ECD-Fc (10 μg/mL, ECD) and Human-Fc (10 μg/mL). Protein band intensity quantification of phosphorylated human Tau at sites S262 (**P**) and S396 (**Q**) in Wt mice normalized with total human Tau and expressed as fold change relative to non-treated control (0). Data are represented as the mean ± SEM, n=6 animals. All statistical comparisons were performed using one-way ANOVA and Tukey’s test. Statistical significance: Statistical significance: *P<0.05, **P<0.01, ***P<0.001, ****P<0.0001.

Amyloid beta induces Tau hyperphosphorylation, neurite degeneration and neurotoxicity, leading to AD [51–53]. The neurotoxic activity of Aβ is in part through p75^NTR^ [54–57]. p75^NTR^ is also a receptor to neurotrophins such as proBDNF. It is reported that proBDNF binds to p75^NTR^ with greater affinity than mature neurotrophins [58, 59], inducing apoptosis by activating the receptor complex, p75^NTR^ and sortilin [58]. Therefore, we investigated whether Aβ and proBDNF has a role in p75^NTR^-dependent Tau phosphorylation using SH-SY5Y-APP cells and primary cortical neurons, isolated from pR5 and pR75KO mice. We treated SH-SY5Y-APP with Aβ_42,_ proBDNF and a p75^NTR^ antagonist, p75ECD-Fc (ECD). SH-SY5Y-APP cells were exposed to 1.0 μM Aβ, 30 ng/mL proBDNF (proB) and 30 ng/mL proBDNF with 10 μg/mL p75ECD-Fc (proBE) for 24 hours. The levels of phosphorylated Tau were determined by western blotting (Figure 5A). Amyloid beta treatment significantly increased hyperphosphorylation of human Tau at sites S262 (Figure 5B, p=0.0014), S396 (Figure 5C, p=0.0053) and AT8 (Figure 5D, p=0.0049) compared to non-treated SH-SY5Y-APP cells. ProBDNF treatment increased S396 (Figure 5C, p=0.0093) and S202/T205 (AT8) (Figure 5D, p=0.0047) compared to non-treated SH-SY5Y-APP cells. On the other hand, p75ECD-Fc treatment does not seem to reduce proBDNF-induced phosphorylation of Tau in these cells. Next, we also treated cortical neurons from pR5 and pR75KO mice with different doses of Aβ (0.3 μM, 2.0 μM) (Figure 5E). Amyloid beta increased S262 levels in pR5 cortical neurons at both concentrations at 0.3 μM compared to Wt (Figure 5F, p=0.0026) but not in cortical neurons of pR75KO mice (Figure 5G). Similarly, proBDNF increased S262 (p=0.0001) (Figure 5I) and S396 (Figure 5J, p=0.0260) in pR5 neurons but not pR75KO neurons (Figure 5K, 5L) compared to non-treated cortical neurons. The addition of p75ECD-Fc attenuated S262 levels (Figure 5I, p=0.0016). However, the phosphorylation at S396 after proBDNF and p75ECD-Fc seemed to be reduced but did not reach statistical significance (p=0.0612) in pR5 neurons (Figure 5J). We also investigated the effect of proBDNF on mouse Tau phosphorylated at S396 using Wt neurons and we also found that proBDNF increased mouse Tau phosphorylation at S396 (p=0.0447) while the addition of p75ECD-Fc reversed the effect of proBDNF (p=0.0194) (Figure 5M, 5N). This indicates that Aβ- and proBDNF-induced Tau phosphorylation is specifically through p75^NTR^. To determine if p75ECD-Fc alone affects the basal level of phosphorylated Tau, we exposed pR5 neurons to 10 μg/mL of p75ECD-Fc and Human-Fc (negative control) (Figure 5O). We did not observe any effect on S262 and S396 levels (Figure 5P, 5Q). Overall, these results suggest that p75^NTR^ is required for Aβ and proBDNF-induced phosphorylation of Tau.

### Inhibition of kinases downstream of p75^NTR^ attenuates Aβ-mediated Tau phosphorylation

**Figure 6:**
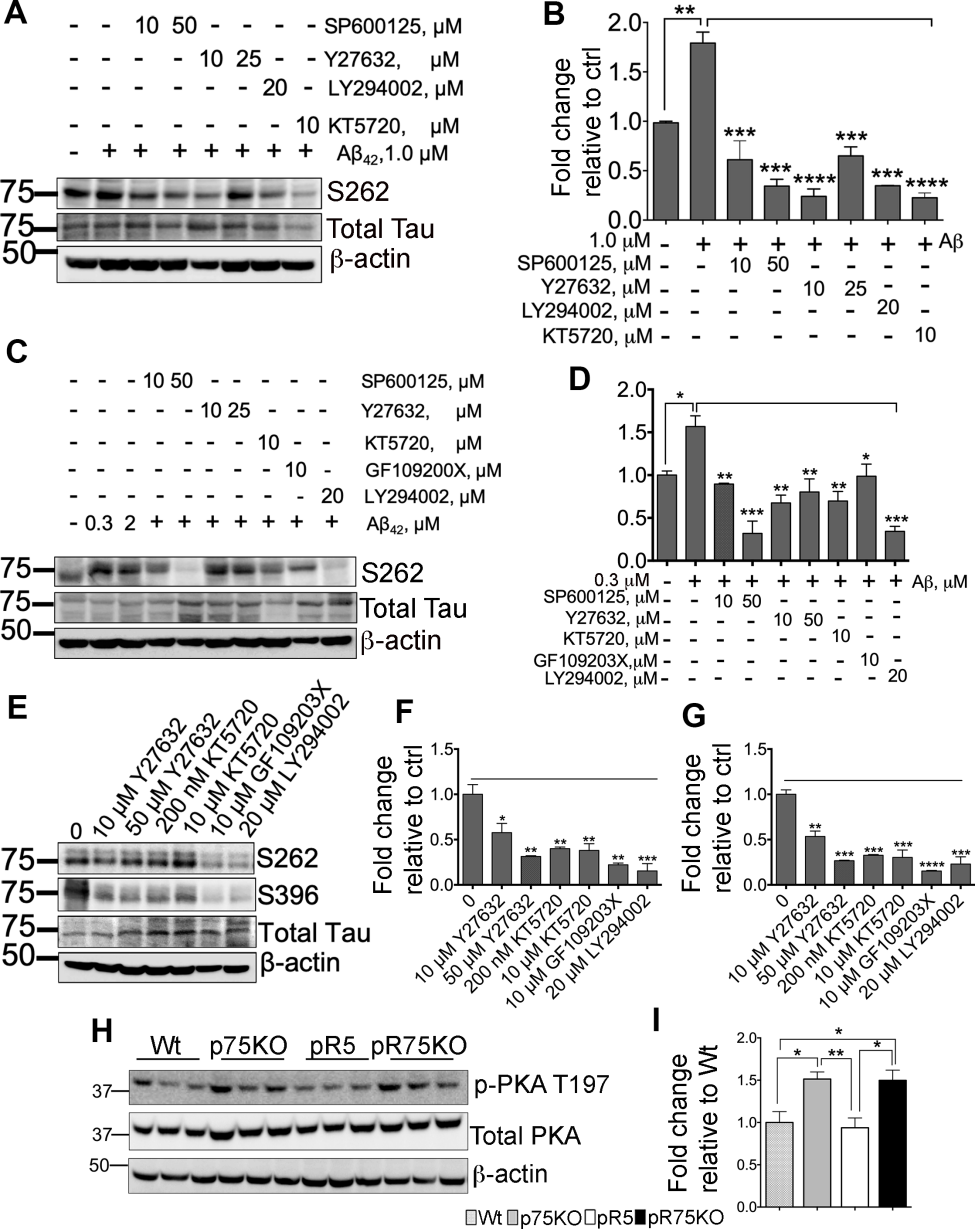
**Various kinase inhibitors attenuated Tau hyperphosphorylation of neurons *in vitro*.** (**A**) Protein blot of phosphorylated human Tau at sites S262 in SH-SY5Y-APP cell line treated with or without Aβ_42_ (1 μM), and subsequently treated with several kinase inhibitors for JNK (SP600125, 10 and 50 μM), ROCK (Y27632, 10 and 25 μM), PI3K (LY294002, 20 μM), and PKA (KT5720, 10 μM) in the presence of Aβ_42_ for 24 hours. (**B**) Protein band intensity quantification of phosphorylated human Tau at site S262 levels in SH-SY5Y-APP cell line. Data are represented as the mean ± SEM, n=3. (**C**) Protein blot of phosphorylated human Tau at sites S262 in primary cortical neurons from pR5 mice treated with or without with Aβ_42_ (0.3 and 2 μM), and subsequently treated with inhibitors for JNK (SP600125, 10 and 50 μM), ROCK (Y27632, 10 and 25 μM), PKA (KT5720, 10 μM), PKC (GF109203X, 10 μM) and PI3K (LY294002, 20 μM) in the presence ofAβ_42_ (0.3 μM)_._ (**D**) Protein band intensity quantification of phosphorylated human Tau at site S262 levels in primary cortical neurons from pR5 mice normalized with total human Tau and expressed as fold change relative to non-treated control (0). Data are represented as the mean ± SEM. Experiment was done in 3 replicates, each replicate has n=12 animals. (**E**) Protein blot of phosphorylated human Tau at sites S262 and S396 in primary cortical neurons from pR5 mice treated with inhibitors for ROCK (Y27632, 10 and 50 μM), PKA (KT5720, 200 nM and 10 μM), PKC (GF109203X, 10 μM) and PI3K (LY294002, 20 μM). Protein band intensity quantification of phosphorylated human Tau at sites S262 (**F**) and S396 (**G**) in primary cortical neurons from pR5 mice normalized with total human Tau and expressed as fold change relative to non-treated control (0) Data are represented as the mean ± SEM. Experiment was done in 3 replicates, each replicate has n=12 animals. (**H**) Protein blot of PKA phosphorylated at site T197 and total PKA in 6 month old mice. (**I**) Protein band intensity quantification of phosphorylated PKA at site T197 normalized with total PKA and expressed as fold change relative to Wt mice. Data are represented as the mean ± SEM, n=6. Statistical comparisons were performed using one-way ANOVA and Tukey’s test. Statistical significance: *P<0.05, **P<0.01, ***P<0.001, ****P<0.0001.

SH-SY5Y-APP cells were used to determine the role of kinases in Aβ-mediated Tau hyperphosphorylation. Cells were subsequently treated with 1.0 μM Aβ_42_ and with various kinase inhibitors for JNK (SP600125), ROCK (Y27632), PI3K (LY294002) and PKA (KT5720) at the indicated concentrations (Figure 6A). Tau phosphorylation at S262 was detected by western blotting and corrected per total human Tau protein. Our preliminary results showed that Aβ treatment significantly increased S262 phosphorylation (p=0.0016), but was reduced when treated by the kinase inhibitors (Figure 6B). This result suggests that Aβ-induced phosphorylation of Tau involves activity of JNK, ROCK, PKA and PKC *in vitro*. We further confirmed this result using pR5 primary cortical neurons. We found that inhibitors for JNK (SP600125: 10, 50 μM), ROCK (Y27632: 10, 50 μM), PKA (KT5720: 10 μM), PKC (GF109203X; 10 μM) and PI3K (LY294002; 20 μM) inhibited Aβ-mediated Tau phosphorylation (Figure 6C, 6D). To determine whether the kinase inhibitors affect the basal level of phosphorylated Tau at sites S262 and S396, we treated pR5 cortical neurons with inhibitors of ROCK (Y27632; 10, 50 μM), PKA (KT5720: 200 nM, 10 μM), PKC (GF109203X; 10 μM) and PI3K (LY294002; 20 μM) (Figure 6E). Interestingly, basal S262 levels (Figure 6F) and S396 (Figure 6G) were also significantly reduced, suggesting that Tau phosphorylation is mediated through these kinases.

A previous report showed that in AD, reduced PKA activation caused by overexpressed calpain resulted in decreased cAMP-response element-binding protein (CREB) function [60]. Decreased CREB is associated with cognitive impairment in AD [60], thus we also investigated whether PKA activity is altered in 6 months old pR5 mice with p75^NTR^ deletion. We found that PKA is activated as shown by increased phosphorylation at T197 [61] in pR75KO mice compared to pR5 mice (Figure 6H), supporting a potential role of p75^NTR^ in the regulation of PKA activity in pR5 mice (Figure 6I). However, at 9 months, PKA activity levels in pR75KO mice were comparable to pR5 (Supplementary Figure 5). This indicates that the increase in PKA activation as a result of p75^NTR^ deletion is not a continuous process and the changes observed may be age-dependent.

### pR5 mice displayed hyperactivity but unaltered cognition

**Figure 7:**
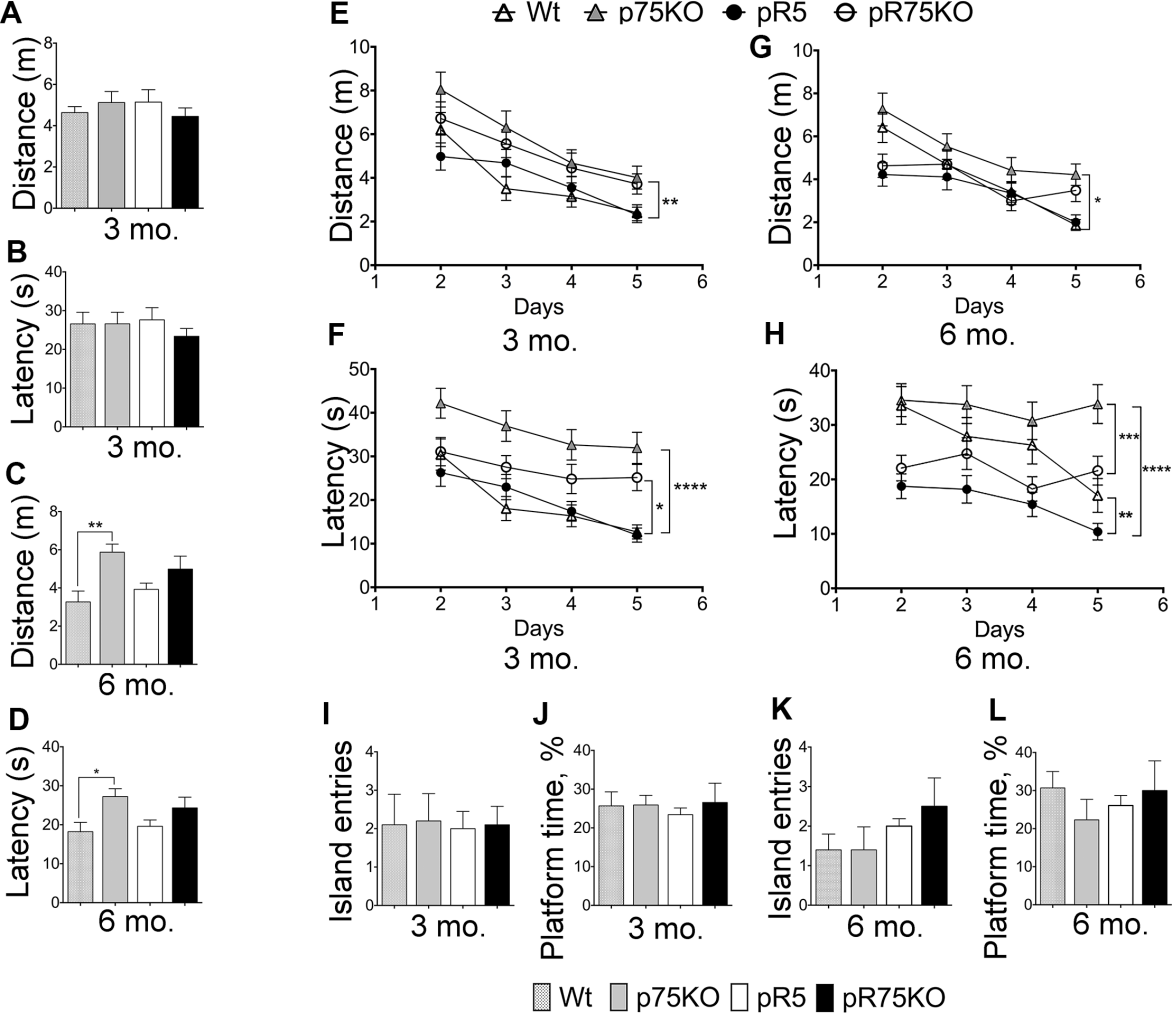
**Deletion of p75^NTR^ reversed hyperactivity in pR5 mice at 6 months.** Wt, p75KO, pR5 and pR75KO mice at 3- and 6-months of age were subjected to MWM test. Performance of mice on Day 1 to locate the visible platform was assessed by measuring total distance travelled in metres (m) and escape latency in seconds (s) at 3 months (**A** and **B**) and at 6 months (**C** and **D**) of age. Performance of mice on training Days 2-5 to locate the platform where it is submerged was assessed by measuring the total distance travelled and the escape latency during training at 3 months (**E** and **F**) and 6 months (**G** and **H**) of age. To determine memory impairment in mice, Probe Test was performed where the number of island entries or platform crossing and the percentage of time spent at the platform area by each mouse, were recorded at training Day 6, at 3 months (**I** and **J**) and 6 months (**K** and **L**) of age. Data are represented as the mean ± SEM, n=12. Statistical comparisons were performed using one-way (Day 1 and Probe Test) or two-way ANOVA (Training) and Tukey’s test. Statistical significance: *P<0.05, **P<0.01, ***P<0.001, ****P<0.0001.

To assess the difference in spatial reference memory among the mouse group, mice were subjected to Morris Water maze test, a commonly used test that relies on an intact hippocampus [62] at 3 and 6 months of age. Wild type and p75KO mice were used as controls for comparison of basal behavior. At the visible platform trial performed on Day 1, escape latency, path-length or total distance, and swimming speed were recorded. 3 months old mice did not show any differences in performance on Day 1 (Figure 7A, 7B, Supplementary Figure 6A). During this trial, mice are not expected to show any difference, however, the phenotypic characteristic of p75KO might account for the difference as these animals have a greater susceptibility to stress [63]. However, at 6 months of age, p75KO mice have early signs of impairment as shown by the longer distance travelled (Figure 7C, p=0.0059) and latency to find the visible platform (Figure 7D, p=0.0343) and slow swimming speed compared to Wt mice (Supplementary Figure 6B, p=0.0013) (one-way ANOVA, Tukey’s post hoc test, p=0.05). In addition, the difference is also because the activity of Wt and pR5 mice were found increased at 6 months as indicated by the reduction in distance travelled and latency time to find the platform (Figure 7A–7D). Most of the p75KO mice tested showed stress-related behavior and had a tendency to float on the water instead of swimming.

Training was done during the next 4 consecutive days with the platform submerged 1 cm below the surface. During the training, 3 months old p75KO mice covered the longest distance compared to Wt (p=0.0010) and pR5 (p=0.0016) while pR5 and pR75KO mice performed similarly to Wt mice (Figure 7E). The latency to find the platform of Wt and pR5 mice were comparable while p75KO (p<0.0001) and pR75KO mice (p=0.0256) had longer latency than Wt mice (Figure 7F). The swimming speeds of all mice groups were similar at 3 months of age (Supplementary Figure 6C). A change in behavior became apparent at 6 months of age. p75KO mice covered significantly longer distance compared to Wt (p=0.0217), pR5 (p<0.0001, not indicated on the graph) and pR75KO mice (p=0.0079, not indicated on the graph) while Wt, pR5 and pR75KO mice covered similar distance (Figure 7G). In addition, p75KO mice took the longest time to find the platform as indicated by the latency time which was significantly longer than Wt mice at 6 months of age (p=0.0355). Wt mice showed similar latency to find the platform in comparison to pR75KO mice (Figure 7H). pR5 mice showed the shortest latency compared to Wt (p=0.0013) and p75KO mice (p<0.0001) while pR75KO mice also showed decreased latency compared to p75KO mice (p=0.0003). Interestingly, pR5 mice were the fastest swimmers and swam significantly faster than Wt (p<0.0001), p75KO (p=0.0003, statistical significance is not shown on the graph to avoid too many symbols) and pR75KO mice (p=0.0015) at 6 months of age (Supplementary Figure 6D). These data suggest p75KO mice showed the greatest cognitive impairment (Figure 7G, 7H) compared to Wt. Interestingly, pR5 mice were faster than Wt with reduced latency time and increased swimming speed (Figure 7H, Supplementary Figure 6D) despite \ covering similar distances as Wt mice (Figure 6G). This type of hyperactivity displayed by pR5 mice is also another evidence of an altered exploratory behavior [62]. However, during the Probe test, no difference in memory was observed among mice groups at both ages (Figure 7I–7L). The memory impairment in our animal models may not be severe enough at these ages to be shown using the MWM Test. Overall, the knockout of p75^NTR^ impairs spatial learning similar to a previous finding [64] as we found that 3 and 6 months old p75KO mice displayed increased distance covered (Figure 7E, 7G) and latency time (Figure 7F, 7H) to find the platform versus Wt and pR5 mice respectively. In addition, the knockout of p75^NTR^ also reverses the hyperactivity observed in P301L Tau model [65], as indicated by the latency time (Figure 7H) and the pattern of the swimming speed in pR75KO which were not significantly different to Wt mice (Supplementary Figure 6D).

## DISCUSSION

The neurotrophin receptor p75^NTR^ has been found to mediate critical pathological conditions in AD, such as (1) neurite degeneration [55, 66], (2) neuronal death via Aβ [54, 67, 68] and via proNGF [55, 69, 70], and (3) increased Aβ production [71, 72]. It has also been shown that the use of antibody directed against the extracellular domain of p75^NTR^ inhibited Aβ-induced neuronal death [54, 55]. The colocalization of p75^NTR^ with phosphorylated Tau suggests that the receptor could potentially induce more signaling towards NFT formation in AD [29]. However, the mechanisms by which p75^NTR^ modulates Tau hyperphosphorylation remain to be further elucidated. By deleting p75^NTR^ in pR5 mice, human Tau hyperphosphorylation was significantly reduced while total human Tau protein expression was unaffected. We have also found that knocking out p75^NTR^ also attenuated kinase activities of GSK3, Cdk5 and ROCK in pR5 mice at 9 months of age. Other kinases such as JNK, PI3K and PKC were also modulated by p75^NTR^ in pR5 mice. We also found that p75^NTR^ could also have a role at post-synaptic sites in younger pR5 mice.

### p75^NTR^ regulates Tau phosphorylation and kinase activities

We have found that the genetic reduction of p75^NTR^ in pR5 mice resulted in the significant reduction human Tau hyperphosphorylation. The reduction of human Tau phosphorylation in pR75KO mouse model as shown by reduced phosphorylation at Tau sites S262, S396 and S202/T205 (AT8) compared to pR5 mice suggests that p75^NTR^ is a key receptor mediating this process. This process is regulated by several kinases such as GSK, Cdk5, ROCK and potentially by JNK, PI3K, PKA and PKC. In pR5 mice at 9 months of age, the level of inactive GSK3β was elevated after p75^NTR^ deletion. GSK3 kinase is a major kinase that phosphorylates Tau [73]. In previous studies, GSK3α and GSK3β were found to induce PHF-type hyperphosphorylation of Tau [74, 75]. GSK3β transgenic animals have also displayed increased Tau hyperphosphorylation and neurodegeneration [76]. We have also recently shown that the exposure of hippocampal neurons to proNGF, a ligand of p75^NTR^, could reduce pS9-GSK3β levels and increase Tau phosphorylation [77], which were also reduced in our pR75KO model. Other p75^NTR^ ligand such as Aβ have been shown to induce GSK3β activation, which then activated Tau hyperphosphorylation and resulted in neuronal death at the hippocampus [76]. Older pR5 mice also have decreased levels of active RhoA-GTP and decreased p25/p35 ratio. The activation of ROCK phosphorylates Tau at threonine 245 (Thr245), Thr377 and Ser409 [44], depends on the presence of RhoA-GTP. p75^NTR^ interacts and activates RhoA, from its GDP-bound form to its GTP form, by displacing it from Rho-GDP dissociation inhibitor [45]. The absence of the full-length p75^NTR^ in pR75KO mice resulted in the decreased conversion of RhoA-GDP to RhoA-GTP and subsequently ROCK activation. In addition, we found that Cdk5 activity is reduced. The reduced Cdk5/p25 ratio in pR75KO animals also potentially supports the role of p75NTR in Cdk5 regulation of Tau hyperphosphorylation. A recent study has elucidated that the interaction of p35 with p75^NTR^ enhanced p25/ Cdk5 signalling by promoting the dephosphorylation of p35 [78]. Thus, the knockout of p75NTR potentially suppresses the activity of kinases that are responsible for phosphorylating Tau.

Inhibitors for kinases that are downstream of p75^NTR^ can also block the Aβ-mediated hyperphosphorylation of Tau, similar to p75ECD. We showed that kinase inhibitors of JNK, ROCK, PKA, PKC and PI3K significantly reduced Aβ-induced and basal Tau phosphorylation. JNK activation leads to apoptosis, Tau hyperphosphorylation and amyloid plaque formation in AD [15]. The JNK/p38 pathway regilated by Aβ activates p53 and translocates nuclear factor-kappaB (NF-κB) via p75^NTR^ [79–81], leading to Tau pathology [82, 83]. Thus, in the absence of full-length p75^NTR^ in pR75KO mice, Tau hyperphosphorylation via JNK is reduced or prevented. PKA activity in AD phosphorylates Tau early during paired helical filament formation [84]. On the other hand, PKA activity could also prevent Tau hyperphosphorylation as it can physically associate and phosphorylate GSK3 after cAMP activation [85]. This is further supported by our *in vivo* results showing that PKA activity is increased in p75KO and pR75KO mice; these results are consistent with the elevated phosphorylated GSKβ-pS9 (inactive kinase) detected in both strains at 6 months of age, which agrees with a recent study demonstrating that the deletion of p75^NTR^ resulted in the dissociation and activation of the catalytic subunit of PKA [86]. While GSK3β activity did not increase in pR5 mice compared to Wt, it was evident that p75^NTR^ plays a role in its activation and in regulating PKA activity which is upstream of GSK3β and involved in GSK3β phosphorylation/inactivation. This explains the upregulation of PKA activity in p75KO and pR75KO mice at 6 months shown in Figure 6I and the subsequent increase in phosphorylated GSK3β of these mice at 9 months (Figure 4G). In a previous study, the inhibition of PI3K and PKC resulted in over-activation of GSK3β *in vivo*, leading to Tau hyperphosphorylation and spatial memory impairment [87]. However, several studies showed opposing results on the role of PKC in GSK3β. In another study, PKC partially inhibited GSK3β-induced phosphorylation of Tau at the S202/T205 (AT8) and Thr181 sites, but enhanced the phosphorylation of Tau at Thr231 [88, 89]. Our result confirmed that PKC inhibition in cells resulted in reduced Tau phosphorylation. The activation of PI3K/Akt signaling *in vitro* and *in vivo* is known to inactivate GSK3β and cause reduced Tau phosphorylation [18, 20, 21, 90]. It is also suggested that the role of p75^NTR^ for neuroprotection against Aβ occurs in a PI3K-dependent manner [91]. However, our findings contradict this neuroprotective role, rather PI3K inhibition resulted in a decrease in Tau phosphorylation in Aβ-treated cell line and cortical neurons, as well as in non-treated cortical neurons. One likely explanation is that in pR5 mice, the inhibition of PI3K could activate other protein kinases favoring Tau phosphorylation. We did not examine the endogenous level of PI3K/Akt signals in our animal models so further investigation of this kinase would shed light on the role of p75^NTR^ in PI3K/Akt signaling in pR5 mice.

### Synaptic dysfunction was not observed in p75KO and pR5 mice

The human P301L mutation is the key pathogenic factor in apoptosis and astrocytosis in pR5 mouse model [34]. This mutation also leads to increased levels of cleaved caspase-3, which is often co-localized with Tau [92]. Caspase activation has also been reported to truncate Tau, resulting in the generation of Tau aggregates and inducing tangle formation [93]. The reduction of cleaved caspase-3 levels with the knockout of p75^NTR^ in this study in 9 months old pR75KO mice further supports the regulatory function of the receptor’s extracellular domain in activating caspases and mediating neural cell death [94]. Although caspase-3 activity was increased in pR5 mice and subsequently attenuated in pR75KO, we did not see any change in expression of neuronal and astrocyte markers. Since not all cleavage of proteins by caspase-3 will lead to apoptosis, this result is not sufficient to conclude that the P301L human Tau mutation induced neuronal apoptosis in pR5 mouse model at 6 and 9 months of age. However, our work further supports the recent work done by Means JC et al., 2017 [95] showing the increase in caspase-3 activity correlated with the increase in truncated Tau, which is responsible for NFT formation, in aged mice.

In animal models of AD and Tauopathy, synaptic dysfunction and decreased levels of synapse proteins are observed and the increased level of phosphorylated Tau in the synapses has direct correlation with dementia [96]. We found that knockout of p75^NTR^ did not alter the expression of presynaptic proteins SNAP-25 and VAMP2 but increased the post-synaptic protein, PSD-95 in 6 months old pR75KO mice. However, the increase in PSD-95 was not reflected in older animals. Phosphorylated Tau is suggested to physiologically link with PSD-95 through association with Fyn in a complex with N-methyl-D-aspartate receptors (Fyn-NMDR) at the dendrites [97, 98]. When phosphorylated pathologically, Tau shifts from dendrites to post-synaptic sites, inducing neurotoxicity [99]. In pR75KO mice, the increased PSD-95 level is accompanied by reduction in phosphorylated Tau. It is possible that p75^NTR^ contributes to microtubule dynamics in post-synaptic sites potentially through PSD-95, altering Tau function. In another report, hippocampal neurons treated with BDNF showed increased microtubule invasion of dendrites that results in the increased expression of PSD-95, a marker for synaptic strength [100]. The role of p75^NTR^ in microtubule dynamics and Tau phosphorylation in synapses warrant further investigation.

### Tau hyperphosphorylation induced by proBDNF and Aβ is mediated through p75^NTR^

We further investigated whether Tau hyperphosphorylation induced by proBDNF and Aβ is mediated through their interaction with the receptor, p75^NTR^. The function of proBDNF/p75^NTR^ interaction in pR5 mice has not been shown. We found that treatment of SH-SY5Y-APP cells and primary cortical neurons from Wt mice with Aβ and proBDNF increased Tau phosphorylation, but this increase was blocked by p75ECD, confirming that p75^NTR^ mediates phosphorylation through ligand binding. Moreover, using cortical neurons from pR5 and pR75KO mice, we were able to show that Tau phosphorylation is p75^NTR^-dependent (Figure 5H–5J).

### pR5 mice displayed hyperactivity behaviors which were reversed after p75^NTR^ deletion

pR5 mice mimic the Tau pathology observed in human AD such as Tau hyperphosphorylation, somato-dendritic localization of Tau and formation of NFTs [35, 101]. pR5 mice have been demonstrated to develop impairment in spatial reference memory tested by MWM test at 6 and 11 months old [62]. In our study pR5 mice did not present with learning/memory impairment but showed an increase in hyperactivity at 6 months. In fact, learning impairment was more evident in p75KO mice consistent with our previous study [67]. The reason could be that p75^NTR^ is a critical receptor for NGF function, since NGF plays an important role in cognition.

One limitation of this work is that we were not able to determine any cognitive impairment of pR5 mice at 3 and 6 months of age. This could be due to the difference in the breeding and percentage of C57BL6 background in the animals we tested compared to other laboratories and behavioral performance of Wt mice tested. In previous reports, mice harboring P301L Tau mutation at the ages 5 to 7 months old or before the onset of paralysis and cognitive impairment perform better than the control strains, suggesting that the presence of human Tau could initially improve mice cognition [102, 103]. In this study, we also found that pR5 mice tend to perform better than Wt mice however when p75^NTR^ is knocked out, the hyperactivity observed in these mice was reverted to the level comparable to Wt. To better demonstrate the impact of deleting p75^NTR^ in pR5 mice, using older animals >12 months old may be more informative as the activity of GSK3β, and Cdk5 kinases responsible for the human Tau phosphorylation were found attenuated in pR5 mice with p75^NTR^ deletion at 9 months rather than at 6 months.

## CONCLUSIONS

In summary, our results show that p75^NTR^ plays a critical role in human Tau hyperphosphorylation *in vitro* and *in vivo* in pR5 mice. Thus, the new model, pR75KO mice is suitable and useful in understanding the mechanism of Tau hyperphosphorylation in the absence of neurodegenerative ligands such as high levels of Aβ and proneurotrophins. More importantly, this model uncovers the direct link between p75^NTR^ and Tau hyperphosphorylation. The multiple roles of p75^NTR^ in signal transduction makes it a key candidate for drug development aiming to prevent, reduce or reverse Tauopathies.

## MATERIALS AND METHODS

### Animals

To elucidate the role of p75^NTR^ in Tau phosphorylation, pR5 mice with the expression of P301L mutation of human Tau [34, 35] were crossed with p75NTR/ExonIII−/− (p75KO), a model expressing the short form of p75^NTR^, which lacks three of the four cysteine-rich domains with the first cysteine region followed by the stalk, transmembrane and intracellular domain, to generate pR5/p75^-/-^ (pR75KO) mice [104, 105] (Figure 1A). The pR5 mice were provided by Prof. Jurgen Goetz (Queensland Brain Institute, The University of Queensland, Brisbane, Queensland, Australia) [34, 62]. Resulting pR5 mice with p75 heterozygous gene were backcrossed with p75KO mice to derive pR75KO mice. Genotyping of animals was performed by PCR (Figure 1B). The absence of full-length p75^NTR^ (Supplementary Figure 1) and the presence of the transgenic protein, human Tau (Figure 1C) were also shown by immunohistochemistry (IHC) staining. C57BL6 (Wt) and p75KO mice were used as controls in all experiments. Equal number of males and females are used for all experiments except in cultured neurons. Ten animals were used for behavioural studies, 6 animals for immunoblotting and 10 animals for immunostaining. Animals were maintained under standard conditions at 22 °C and a 12 h light:dark cycle with *ad libitum* food and water. Mice procedures were approved by the Animal Ethics Committee of the University of South Australia (U34/14) in accordance to the NHMRC guidelines.

### Immunohistochemistry (IHC)

Hemi-brains were immersion-fixed in 4% paraformaldehyde for 24 h and dehydrated in 30% sucrose before embedding in optimal cutting temperature (OCT) compound. Sections were cut at 30 μm using a microtome-cryostat. Antigen retrieval method was performed using 0.1% SDS in PBS for 10 min followed by blocking in 5% BSA with 0.5% Triton-X in PBS overnight. Different sections were treated with the following primary antibodies: mouse anti-human Tau HT7 (Thermo Fischer Scientific Pty, Australia), rabbit anti-p75 ECD (9650) (a kind gift from Prof. Moses V. Chao, Department of Cell Biology, Skirball Institute, and New York, US). Sections were incubated overnight with primary antibodies at 4°C and further developed with biotinylated secondary antibodies followed by treatment using the ABC Kit (Vector Laboratories, CA, USA). Sections were mounted on gelatin-coated slides, serially dehydrated with ethanol and xylene (3 min 75% ethanol, 2 min 85% ethanol, 2 min 95% ethanol, 2 min 100% ethanol, 2 min 100% ethanol, 2 min xylene), and fixed with DPX mounting medium (Sigma-Aldrich, St Louis, MO, US). Images were obtained using Olympus BX53 Light microscope (Olympus, NSW, Australia).

### Immunoblotting

Frozen brain tissues were powdered in liquid nitrogen using a ceramic mortar and pestle, transferred into pre-weighed homogenization tubes, and homogenized in radioimmunoprecipitation assay (RIPA) buffer (50 mM Tris (pH 7.4), 150 mM NaCl, 1% TritonX-100, 1% sodium deoxycholate, 0.1% SDS, 1% NP-40) containing protease inhibitor cocktail and phosphatase inhibitors (100 mg tissue in 1 mL buffer) using Precellys 24 Tissue Homogeniser (Bertin Technologies, France). Glass beads were added into the tube before the homogenization process. The homogenates were centrifuged at 13,000 rpm for 20 min, at 4°C and the supernatants were collected and subjected to immunoblotting. Protein concentrations were measured in all samples using bicinchoninic acid assay (BCA kit) (Thermo Scientific, Rockford, USA) according to the manufacturer’s instructions. 10 μg of brain protein were separated on SDS polyacrylamide gel electrophoresis and then transferred to nitrocellulose membranes. The membranes were incubated with 5% skim milk (for non-phophorylated proteins) or 5% BSA (for phosphorylated proteins) in Tris-buffered saline containing 0.1% Tween-20 (TBST) for 1 h at room temperature. The blots were incubated with primary antibodies overnight at 4°C and washed with TBST for 10 min 3 times. Immunoblots were then incubated with corresponding secondary antibodies for 1 h at room temperature. The immunoblots were developed using enhanced chemiluminescence (ECL) detection reagent kit (Amersham, UK) and visualized using ImageQuant LAS 4000 imaging system (GE Healthcare, UK). Band densities were quantified using ImageJ software [106] relative to the density of control samples. Primary antibodies used were as follows: mouse anti-human Tau HT7 and mouse anti-pan Tau, Tau5 (Thermo Fischer Scientific Pty, Australia); phosphorylated Tau anti-S262 and anti-S396 (Abcam, VIC, Australia); mouse anti-Phospho-PHF-Tau pSer202+Thr205 monoclonal antibody (AT8) (Cat.No. MN1020, CiteAb, UK); sheep anti-human Tau (Antibody Technology Australia, Australia); cleaved caspase-3, rabbit anti-GSK3α/β (Ser21/9), rabbit anti-GSK3β pS9, rabbit anti-p25/35, anti-phosphorylated PKA T197, and anti-total PKA C-α (Cell Signalling Technology, QLD, Australia); rabbit affinity purified anti-ChAT was from Dr**.** John Oliver (Centre for Neuroscience, Department of Human Physiology, Flinders University); mouse anti-tyrosine hydroxylase (TH) (Sigma-Aldrich, St Louis, MO, US) anti-neuronal nuclei antigen (anti-NeuN) (Merck Millipore, VIC, Australia); rabbit anti-glial fibrillary acidic protein (anti-GFAP) (DAKO, Denmark); rabbit anti-vesicle-associated membrane protein 2 (VAMP2) and rabbit anti-synaptosomal-associated protein 25 (SNAP25) (OSS00035W, Osenses, Australia); anti-postsynaptic density protein 95 (PSD-95) (Sigma-Aldrich, St Louis, MO, US);and mouse monoclonal anti-RhoA-GTP and rabbit anti-total RhoA (New-East Biosciences, Malvern, Pennsylvania, US). Mature BDNF was from Santa Cruz Biotechnology (USA). Anti-proBDNF was a kind gift from Prof. Ru-Ping Dai (Department of Anesthesiology, the Second Xiang-Ya Hospital of Central South University). Anti-β-actin (Sigma-Aldrich, St Louis, MO, US) or anti-glyceraldehyde-3-phosphate dehydrogenase (GAPDH) (Osenses, Australia) were used as loading controls.

### Oligomeric Aβ preparation

In this study, oligomeric Aβ_42_ was prepared as previously described [107]. Briefly, 1 mg of synthetic Aβ_42_ peptide (Sigma-Aldrich) was dissolved in 1 mL 1,1,1,3,3,3-hexafluoro-2-propanol (HFIP) (Sigma-Aldrich) and aliquoted in smaller volumes with the desired stock concentration (e.g. 5 or 20 μg). HFIP was evaporated completely in a fume hood and the Aβ pellets stored at -80°C until use. To prepare the oligomeric species, Aβ_42_ was dissolved in cold DMEM at 25 μM, vortexed vigorously and incubated at 4°C for 24 h.

### Cell and primary neuronal cultures

SH-SY5Y-APP cells were from Prof. Nigel Hooper (Institute of Molecular & Cellular Biology, University of Leeds). Cells were grown in Dulbecco modified eagle’s medium (DMEM) (Invitrogen, Mulgrave, VIC, Australia) supplemented with 10% FBS and 2 mM L-glutamine and 1% penicillin/streptomycin and incubated at 37°C in a humidified atmosphere of 95% air and 5% CO_2_. After overnight seeding of SH-SY5Y-APP cells in 6-well plates (1x10^6^/well) (Invitrogen, Mulgrave, VIC, Australia), cells were co-treated with 1.0 μM Aβ_42_ and kinase inhibitors: JNK inhibitor (SP600125), ROCK inhibitor (Y27632) and, PKA inhibitor (KT 5720) all obtained from Sigma-Aldrich (St Louis, MO, US), PI3K-Akt inhibitor (LY294002, A.G. Scientific, San Diego, CA) and PKC inhibitor (GF109203X) (Tocris Bioscience, UK) for 24 h. Primary cortical neurons were obtained from Wt, pR5 and pR75KO pups aged 0-1 day. Cortical neurons were separated from cortices in DMEM on ice by using trypsin digestion at 37°C for a maximum of 20 min with agitation every 5 min. Digestion was stopped by adding 15% fetal bovine serum (FBS). Cell debris were allowed to settle for 5-10 min, afterwards, cell suspension was collected and centrifuged at 2000 rpm, 2 min at 4°C. Cells were re-suspended in Neurobasal medium (Invitrogen, Mulgrave, Australia) supplemented with 2% B27, 1% penicillin/streptomycin and 2 mM L-glutamine. The cortical neurons were then seeded on PDL-coated 6-well plates at 1.0 X10^6^ cell per well for Western blotting. Cells were lysed in RIPA buffer. Neurons were treated at DIV4 (4 days in vitro), when primary neurons are considered mature, with either 1.0 μM Aβ_42_, 50 ng/ml of proBDNF (Virovek, USA) or co-treated with p75ECD-Fc protein for 24 h. Supernatants were collected after centrifugation of the lysates at 14,000 rpm for 20 min at 4°C. Protein concentration was measured using BCA Assay Kit (Thermo Fischer Scientific, Rockford, USA). Equal amounts of protein mixed with 5 x SDS Loading buffer (0.2 M Tris-HCl, pH 6.8, 10% w/v SDS, 20% v/v glycerol, 5% β-mercaptoethanol, 0.05% w/v bromophenol blue) were boiled for 5 min and stored at -20 °C until analysis.

### Behavioral analysis

Morris Water Maze (MWM) test was performed according to published protocols with minor modifications [108, 109]. The test was conducted using a black circular pool with a diameter of 100 cm and height of 60 cm filled with water to provide a depth of 21 cm. A non-toxic tempera paint powder (Eckersley’s Art and Craft, Adelaide, SA) was used to make the water opaque. The water temperature was maintained at 22 ± 1°C. The tank was surrounded by a set of spatial cues [110]. The test consisted of one-day pre-training phase with 4 trials, 4 days hidden platform trial with 4 trials and a probe test with single trial. During the pre-training phase, the platform fixed in the designated platform quadrant was placed 1 cm above the water level with a red flag to increase its visibility. Mice were allowed to swim for 60 sec for each trial. If the mouse failed to find the platform within the allotted time, mice were gently guided towards the platform or placed gently onto the platform for additional 20 sec. Mice that found the platform were allowed to remain for 5 sec on the platform before returning them to their cages. During the platform trial, the platform was immersed 1 cm below the water level and similar steps performed during the pre-training were done. During the probe test, the platform was removed and the mice starting position was at the furthest position from the platform and the mice were allowed to swim freely for 60 sec. The performance in all tasks was video-recorded and analyzed by a computer-based video tracking system and image analyzing software, ANY-maze (Stoelting, Co., Wood Dale, IL, USA). In platform trials, distance of path from the start location to the platform (in centimeters), latency of the time taken to reach the platform from the start location (in seconds) were measured, while in probe trials quadrant time (percentages of time spent in the platform quadrant) and platform crossings (the number of times that the mice crossed the exact location of the platform) were measured.

### Statistical analyses

All data were presented as mean ± SEM. A majority of the western blot data was analysed using one-way ANOVA followed by Tukey’s post-hoc test, or Dunnet’s test when applicable. When comparing two groups, two-tailed unpaired t-test was also utilized. For behavioral phenotyping result, test was evaluated using either one-way ANOVA or two-way ANOVA (factors: genotype, and treatment) with Tukey’s post-hoc test. Significance was set at p <0.05. For all figures, *p is <0.05, **p is <0.01, ***p <0.001 and ****p is <0.0001.

## Supplementary Material

Supplementary Figures
