# Supplementary material for: Knockout of p75 neurotrophin receptor attenuates the hyperphosphorylation of Tau in pR5 mouse model
Source: Aging (Albany NY). 2019 Sep 3;11(17):6762–91. doi: 10.18632/aging.102202 (PMC6756909; doi:10.18632/aging.102202)
Supplement: Supplementary Figures [file aging-11-102202-s001.pdf]

SUPPLEMENTARY FIGURES

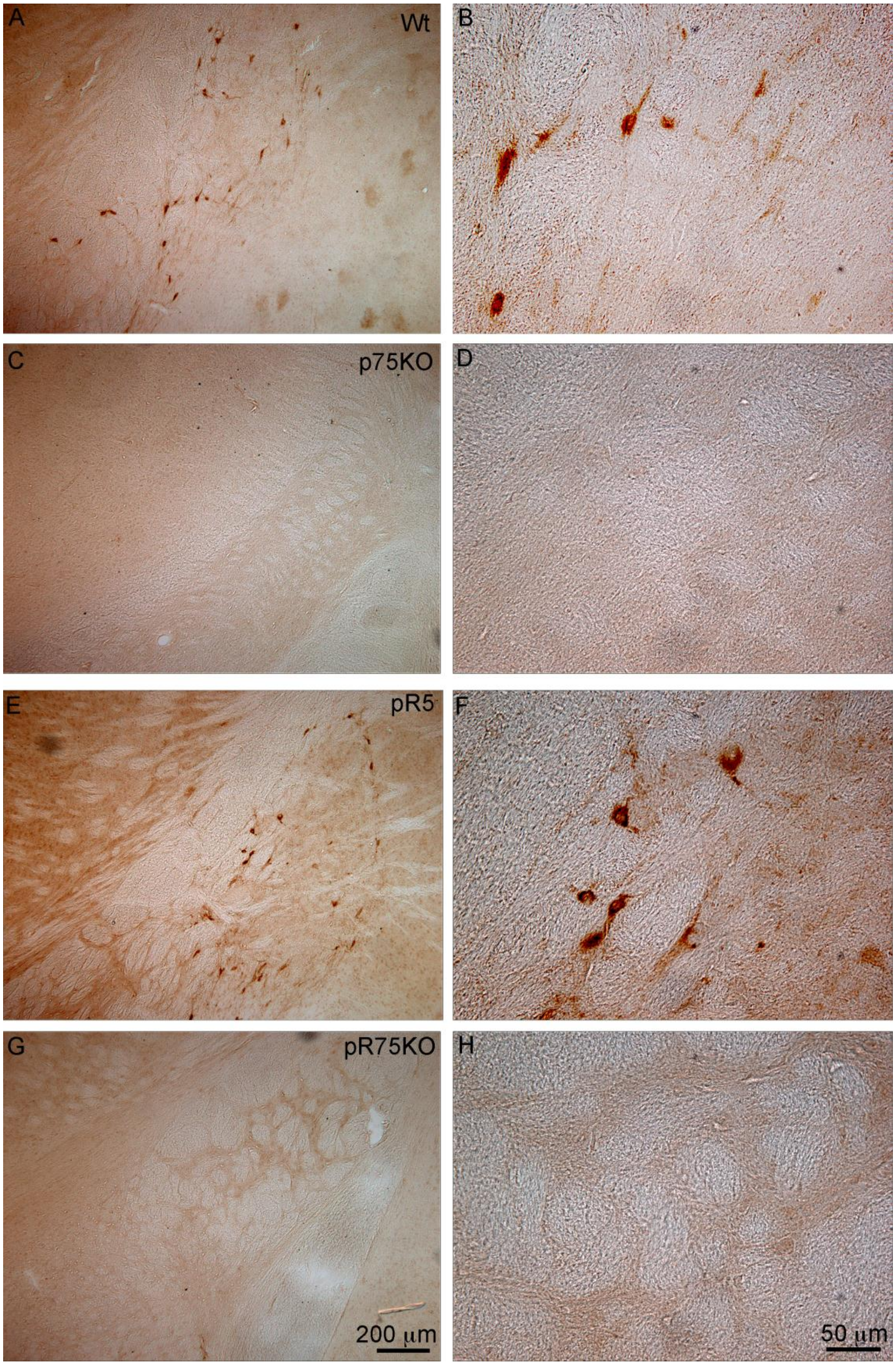

Supplementary Figure 1. p75^{NTR} expression in mice. p75^{NTR} expression detected by p75^{NTR} ECD (9650) in (**A**-**B**) wild type (Wt), (**C**-**D**) p75KO, (**E**-**F**) pR5 and (**G**-**H**) pR75KO mice was mainly observed in the substantia nigra (SN) of Wt and pR5 mice. Scale bar = 200, 50 µm.

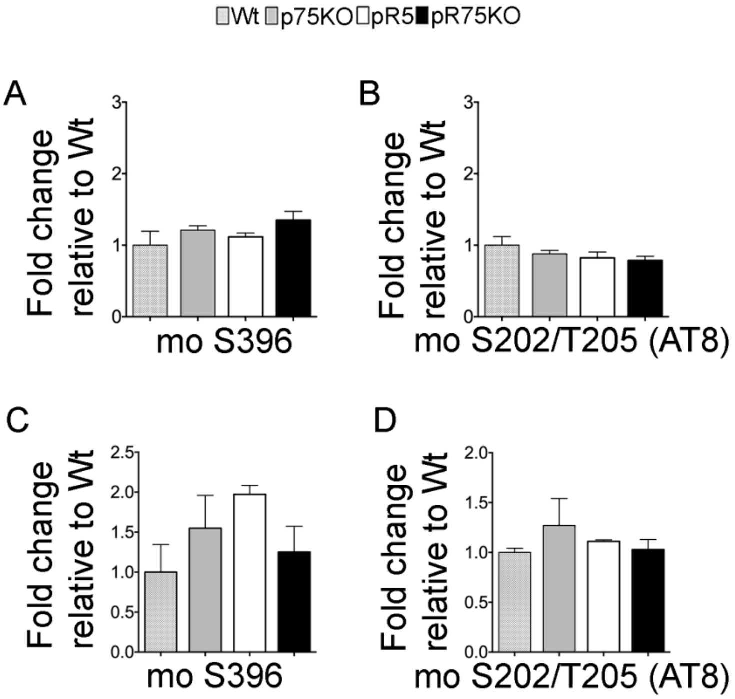

Supplementary Figure 2. Human Tau mutation and p75[NTR] deletion does not affect endogenous mouse Tau phosphorylation. Protein band intensity quantification of mouse pTau at S396 and at S202/T205 (AT8) of Wt, P75KO, pR5 and pR75KO mice at 6 months (Data are represented as the mean ± SEM, n=6) (**A** and **B**) and at 9 months (Data are represented as the mean ± SEM, n=3) (**C** and **D**). Statistical comparisons were performed using one-way ANOVA and Tukey's test. Statistical significance: **P<0.05, **P<0.01*, ****P<0.001, ****P<0.0001*.

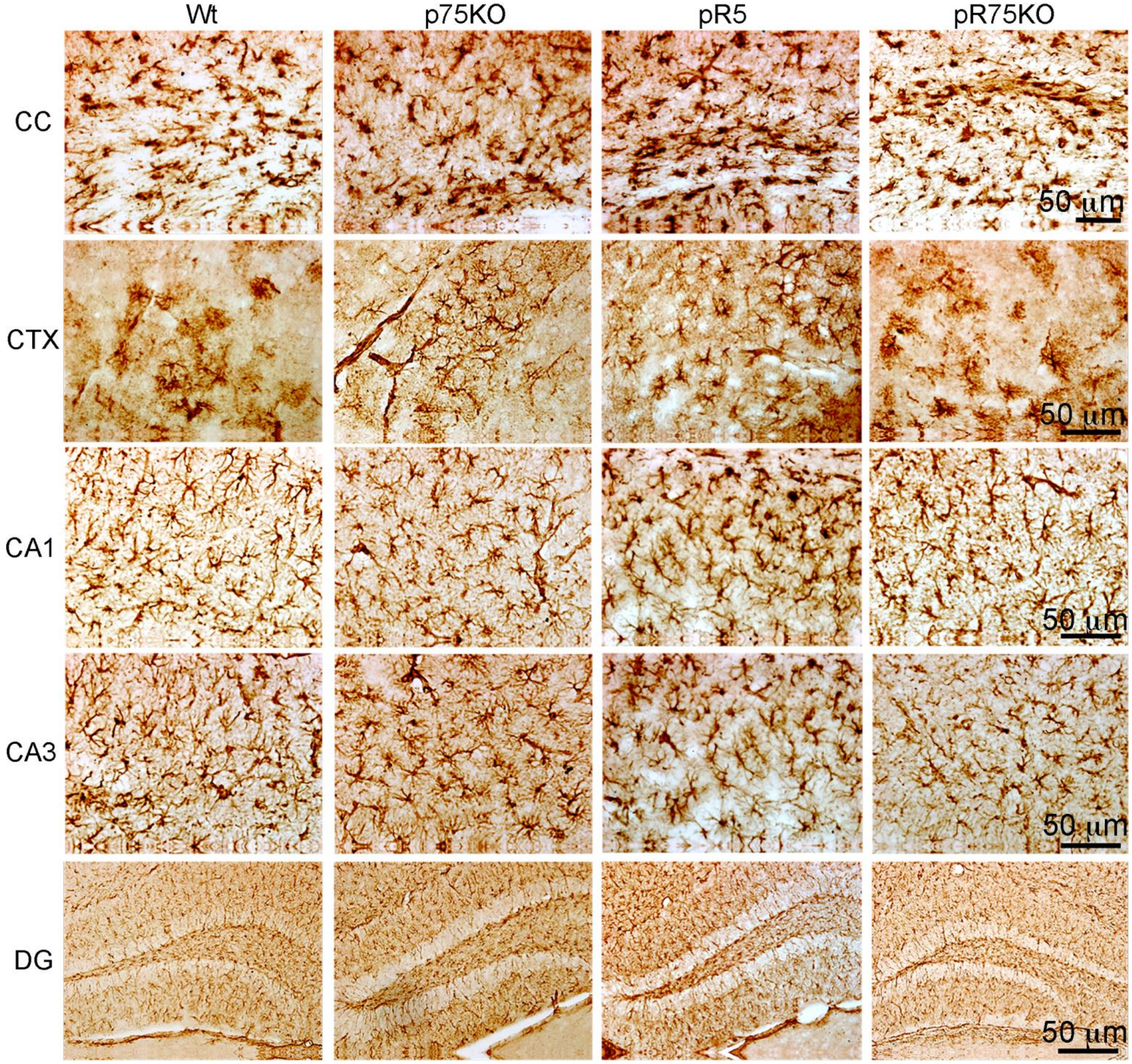

Supplementary Figure 3. Astrocyte staining patterns were similar across all strains at 6 months. Brain sections from 6 months old mice were subjected GFAP IHC-DAB as GFAP is an astrocyte marker. Images were taken from the corpus collosum (CC), cortex (CTX), hippocampus regions (CA1, CA3) and dentate gyrus (DG) in brain sections of Wt, p75KO, pR5 and pR75KO mice. Scale bar = 50 µm.

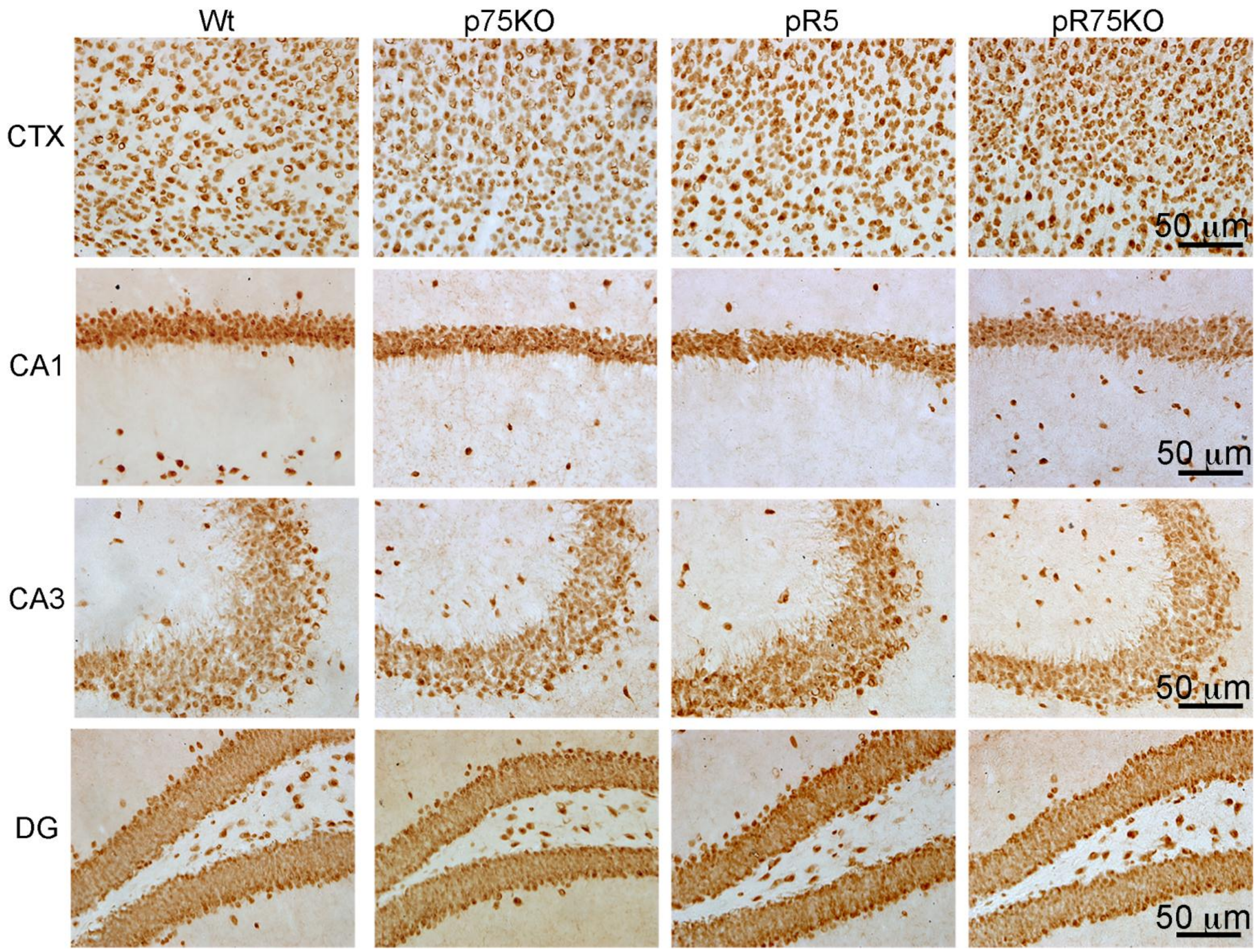

Supplementary Figure 4. Mature neurons staining were similar across all strains at 6 months. Brain sections from 6 months old mice were stained with neuronal nuclei antigen marker, NeuN using IHC-DAB. Images were taken from the cortex (CTX), hippocampus regions (CA1, CA3) and dentate gyrus (DG) in brain sections of Wt, p75KO, pR5 and pR75KO mice. Scale bar = 50 µm.

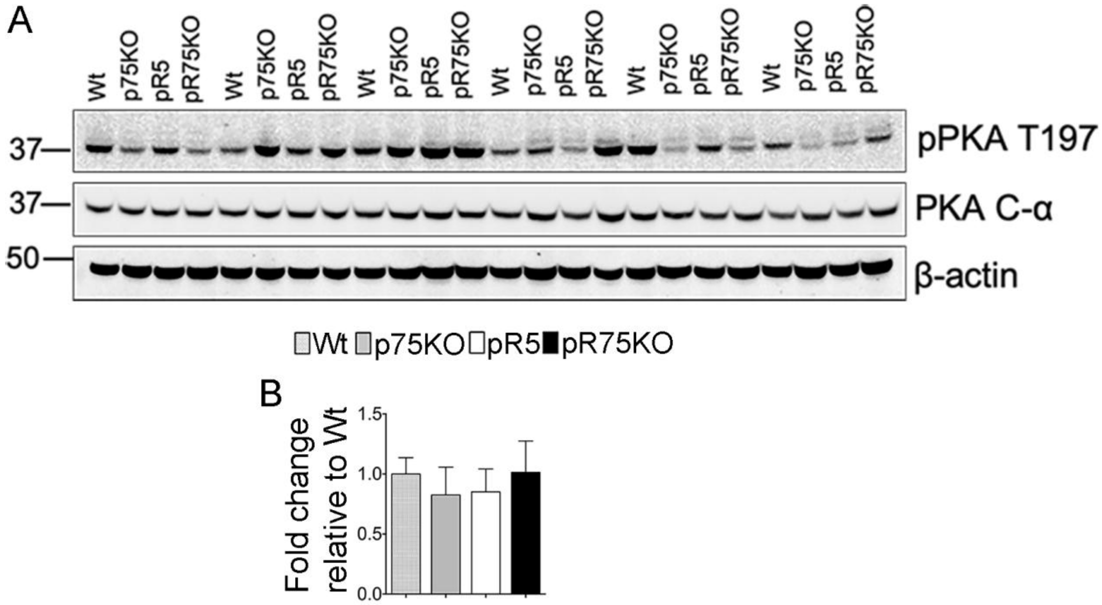

Supplementary Figure 5. PKA activity in 9 months old mice. (**A**) Protein blot of PKA phosphorylated at site T197 and total PKA in 9 months old mice. (**B**) Protein band intensity quantification of phosphorylated PKA at site T197 normalized with total PKA and expressed as fold change relative to Wt mice. Data are represented as the mean ± SEM, n=6. Statistical comparisons were performed using one-way ANOVA and Tukey's test. Statistical significance: *P=0.05*

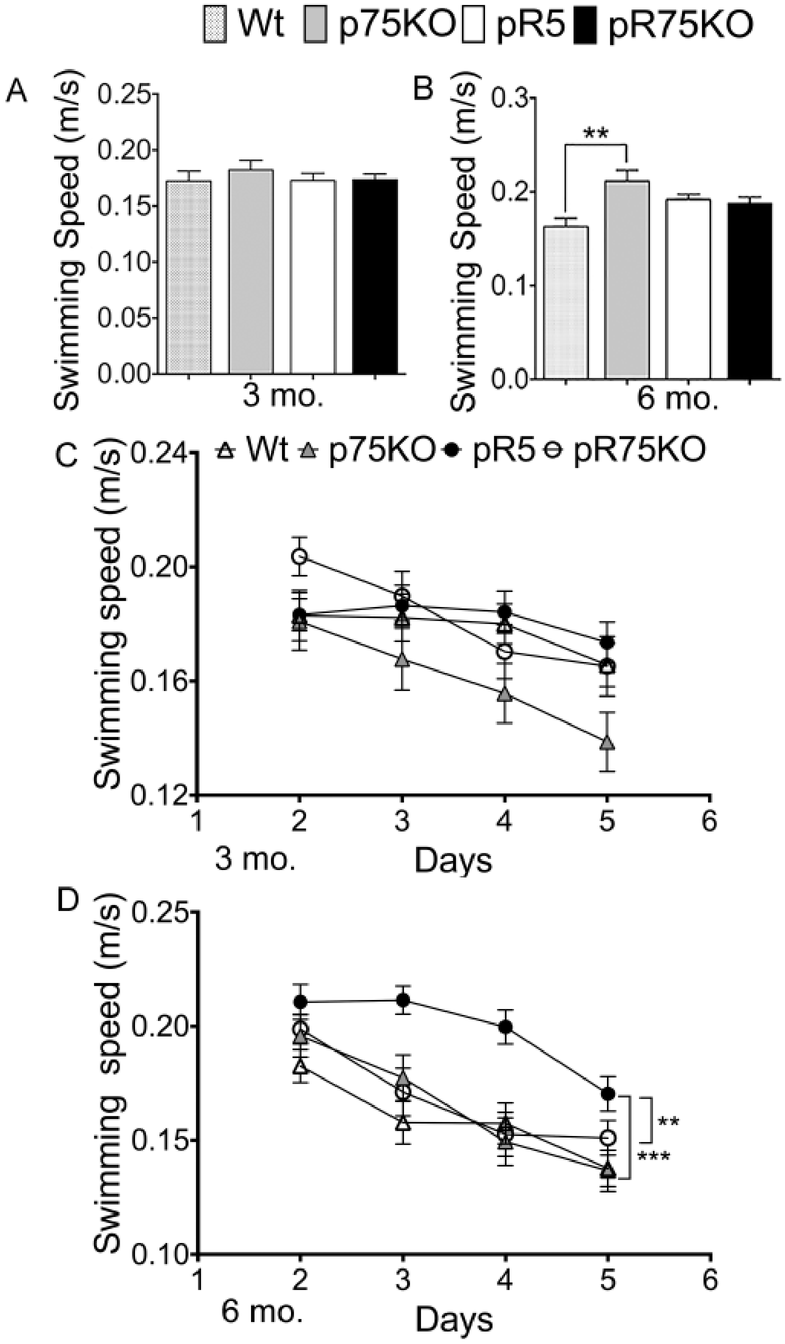

Supplementary Figure 6. Swimming speeds of mice – Morris Water Maze test. Wt, p75KO, pR5 and pR75KO mice at 3 and 6 months of age were subjected to MWM test. Swimming speeds (m/s) of mice on Day 1 to locate the visible platform were assessed at 3 months (**A**) and at 6 months (**B**) of age. Swimming speeds (m/s) of mice on training Days 2-5 to locate the platform where it is submerged were assessed at 3 months (**C**) and 6 months (**D**) of age. Data are represented as the mean ± SEM, n=12. Statistical comparisons were performed using one-way (Day 1 and Probe Test) or two-way ANOVA (Training) and Tukey's test. Statistical significance: **P<0.05, **P<0.01, ***P<0.001, ****P<0.0001*.
